# Which strategies support the effective use of clinical practice guidelines and clinical quality registry data to inform health service delivery? A systematic review

**DOI:** 10.1186/s13643-022-02104-1

**Published:** 2022-11-09

**Authors:** Kathy Dempsey, Caleb Ferguson, Adam Walczak, Sandy Middleton, Christopher Levi, Rachael L. Morton, on behalf of The Australian Health Research Alliance (AHRA) Health System Improvement and Sustainability Working Group members, Rachael Morton, Katherine Boydell, Megan Campbell, Alan Cass, Jed Duff, Catherine Elliott, Gary Geelhoed, Angela Jones, Wendy Keech, Vikki Leone, Danny Liew, Ecushla Linedale, Chips Mackinolty, Lisa McFayden, Sarah Norris, Helen Skouteris, David Story, Rowena Tucker, John Wakerman, Lauren Wallis, Tamsin Waterhouse, John Wiggers

**Affiliations:** 1 Faculty of Medicine and Health, NHMRC Clinical Trials Centre, The University of Sydney, Camperdown, NSW 2050 Australia; 2 University of Western Sydney, Penrith, Australia; 3 Sydney Partnership for Health, Education, Research and Enterprise (SPHERE), University of NSW, Kensington, Australia; 4 Nursing Research Unit, Australian Catholic University, Sydney, Australia

## Abstract

**Background:**

Empirical evidence suggests data and insights from the clinical practice guidelines and clinical quality registries are not being fully utilised, leaving health service managers, clinicians and providers without clear guidance on how best to improve healthcare delivery. This lack of uptake of existing research knowledge represents low value to the healthcare system and needs to change.

**Methods:**

Five electronic databases (MEDLINE, Embase, CINAHL, Cochrane Central and Cochrane Database of Systematic Reviews) were systematically searched. Included studies were published between 2000 and 2020 reporting on the attributes, evidence usage and impact of clinical practice guidelines and clinical quality registries on health service delivery.

**Results:**

Twenty-six articles including one randomised controlled trial, eight before-and-after studies, eight case studies/reviews, five surveys and four interview studies, covering a wide range of medical conditions and conducted in the USA, Australia and Europe, were identified. Five complementary strategies were derived to maximise the likelihood of best practice health service delivery: (1) feedback and transparency, (2) intervention sustainability, (3) clinical practice guideline adherence, (4) productive partnerships and (5) whole-of-team approach.

**Conclusion:**

These five strategies, used in context-relevant combinations, are most likely to support the application of existing high-quality data, adding value to health service delivery. The review highlighted the limitations of study design in opportunistic registry studies that do not produce clear, usable evidence to guide changes to health service implementation practices. Recommendations include exploration of innovative methodologies, improved coordination of national registries and the use of incentives to encourage guideline adherence and wider dissemination of strategies used by successful registries.

**Supplementary Information:**

The online version contains supplementary material available at 10.1186/s13643-022-02104-1.

## Background

Clinical practice guidelines (CPGs) have long been used to inform best clinical practice by compiling the latest, high-quality evidence on the diagnosis, treatment and management of serious acute and chronic illnesses. In Australia, CPGs are generally condition specific and written by clinicians and experts, including consumers.

Clinical quality registries (CQRs) are a more recent advancement, systematically monitoring the quality of healthcare by using the collected data to identify benchmarks and variation in health outcomes. Variations are fed back to clinicians to inform clinical practice, thereby creating a feedback loop, as a mechanism for continuous quality improvement [1].

The growth in the development and acceptability of CQRs was underpinned by the Learning Healthcare Systems (LHS) framework, first outlined by the former United States Institute of Medicine (IoM) in 2006 [2]. Inherent in the LHS is the sequential, circular model where research influences practice and practice influences research [3]; the CQR feedback process is an integral element in the LHS framework, enabling the change management domain by supporting the process of continuous quality improvement (QI). The Australian Commission on Safety and Quality in Health Care (ACSQHC) has highlighted the critical function of CQRs to deliver QI to our health services [1, 4–6].

CQRs function to inform, monitor and report on the implementation of QI measures, aiming to reduce variation and extend best practice care to as many patients as possible, delivering more equitable outcomes [7]. Registries that collect data without benchmarking it against best practice (either through CPGs or high-quality clinical settings) will not have the knowledge to learn from the data and improve quality outcomes. Data entered into the CQR must be locally relevant with implementation supported by those “who know how to initiate, perform, and evaluate quality improvement, and who have the resources to do so” [8].

The change management or implementation phase of the LHS is often less well managed [3, 9], meaning practice is not informed by the relevant outcome data within a reasonable time frame. There are many possible explanations for this, including segregated and siloed groups responsible for knowledge generation, knowledge translation and patient care, the passive role of health consumers and challenges of maintaining communication across silos [10].

Despite there being an abundance of CPGs and CQRs, empirical evidence suggests they are not being used to their full potential [9, 10]. Questions remain about the exact methods and models successful CQRs use to facilitate LHS activities, leaving health service managers, clinicians and other healthcare providers without clear guidance on how best to utilise data and insights from these sources to improve healthcare delivery. An earlier systematic review examined the impact of CQRs as an intervention for improving health outcomes, with the primary interest being mortality or survival [11]. It suggested three reasons why few CQRs had done this well: conflicting roles — quality improvement versus data storage; conflicting use of registry data — for reporting to government and research bodies, but not for assessing and benchmarking health services performance; and a reluctance from registry investors to formally assess registry impact through expensive rigorous trial design [11].

This systematic review addresses a broader research question: *which strategies support the effective use of clinical practice guidelines and clinical quality registry data to identify gaps in best practice care and to inform and improve health service delivery?* The deliberate framing of the research question in this way supports a more positive exploratory process, focusing on the benefits of utilising existing research knowledge to promote high value health services practices and policies.

## Methods

### Literature search

Five electronic bibliographic databases were systematically searched using two separate search processes, between 3 and 30 March 2020: MEDLINE, Embase, CINAHL, Cochrane Central and Cochrane Database of Systematic Reviews (the latter to identify other related reviews). The search strategies included studies published from 1 January 2000 to 30 March 2020 or later because the aim was to inform Australia’s developing health translation services with reference to the contemporary theories of learning healthcare systems. Detailed information on the search strategies is available in Additional file 1. Ethics approval was not required for this systematic review of secondary data.

### Selection criteria

Publications were included if published in English, between January 2000 and March 2020, involving participants of any age, and with no restrictions on country of study or type of medical condition. Included publications presented original empirical research on the attributes and/or impact of CPGs/CQRs on health service delivery (positive, neutral or negative). All design types were eligible (including nonexperimental designs documenting registry processes) providing they included specific information on how the evidence/data was used to inform health service delivery. The only exclusion criteria were particular publication types (study protocols, scientific conference abstracts, reviews of previously published original studies, editorials, commentaries, letters and duplicate publications).

### Literature selection

KD worked with Mr. Roderick Dyson, a research librarian at The University of Sydney, to develop the search criteria for the bibliographic database searches. Initial search terms were based on the suggested key words and nominated databases proposed by The Australian Health Research Alliance (AHRA) Health System Improvement and Sustainability Working Group. Mr. Dyson and KD met on several occasions to refine the search criteria, and two separate searches were conducted to ensure as many relevant publications as possible were identified. The specific details of these searches are contained in Additional file 1.

**Fig. 1:**
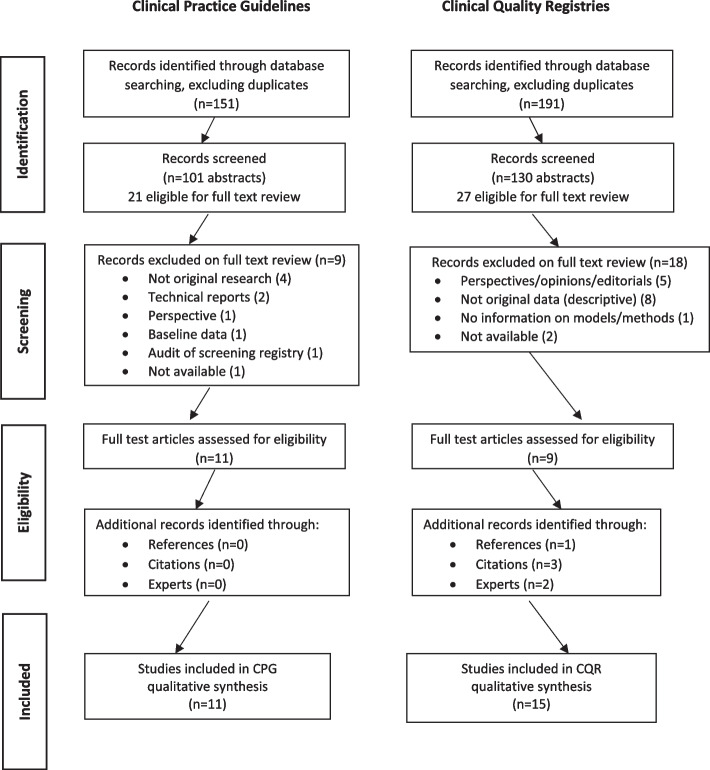
PRISMA diagram [12]

All abstracts identified in the searches were exported to an EndNote version 9 library. KD and CF separately reviewed all abstracts (152 in search 1; 494 in search 2). When exclusion criteria were applied and duplicates removed, this left a total of 231 abstracts for further review (as agreed by KD and CF). Of these, 48 underwent full-text review by both KD and CF via hard copy Microsoft Word documents, and an inter-rater reliability statistic was calculated (see Additional file 1). Differences between reviewers were discussed and a consensus list developed. Reference lists of each included article were searched, and citation searching was also conducted to identify other relevant publications. Academic experts in this field were consulted for additional relevant articles. The selection process is outlined in Fig. 1.

Data on population, intervention, comparator, outcomes and study design (PICOS) is shown in Tables 1 and 2 and included the following: first author, year and location of study (country); population, setting and response rate; intervention/evaluation; comparator; outcome: models/methods used; and study design and time frame. Data abstracted from the publications for the finding tables (Additional files 5 and 6) included the following: first author, year and location of study (country); study objectives, benchmarking; type of guideline; type of registry; healthcare findings; and what works? In addition, CF checked the accuracy of the PICOS tables, and RM checked the formatting of the findings tables; KD is responsible for the content of the findings tables. The quality assessment was undertaken by Ms. Heilok Cheng, a Postgraduate Research Student at The University of Sydney. The accuracy of this assessment was checked by KD.

**Table 1:** Included CPG studies detailing population, intervention, comparator, outcomes and study design (PICOS)

First author, location, year	Population, setting, response rate	Intervention/evaluation	Comparator	Outcome: models/methods used	Study design and time frame
Dyrkorn, Sweden, 2012 [13]	• 744 caesarean section patients • Follow-up > 90% in both groups	∘ Compared infection rates of 197 women pre-intervention with those of 557 women post-intervention • Introduced 5 new measures and strengthened 2 existing measures simultaneously	Infection rates before and after intervention	∘ Established an improvement team ∘ Introduced infection control measures (see Intervention) ∘ Staff received education and awareness programmes in QI • Patients sent questionnaire about wound healing and signs of infection 30 days postoperatively, deep surgical wound infections diagnosed by physician	Before-and-after study (August 2008–2010)
Hendriks, Netherlands, 2012 [14]	∘ 712 patients referred for stable atrial fibrillation (AF) to the outpatients’ department at Maastricht University Medical Centre	∘ AF clinic with nurse-led care consisted of guideline-based, software-supported integrated chronic care supervised by a cardiologist, based on the chronic care model ∘ Primary endpoint was a composite of cardiovascular hospitalization and cardiovascular death	Usual care	∘ Dedicated software used to guide comprehensive management of AF and associated cardiovascular conditions ∘ Software determined individual patient profile based on symptoms, type of AF and stroke risk and proposed the most appropriate management ∘ Patients instructed about rate and rhythm control as well as prophylactic vascular therapy (including strict anticoagulation monitoring) and when to report to the hospital • Psychosocial support and educational interventions repeated at follow-up visits	Randomised controlled trial (RCT). Patients recruited between Jan 2007 and Dec 2008, with a minimum 12-month follow-up
Kamišali´c, Slovenia/Spain, 2018 [15]	• 50 randomly chosen patients registered in the primary care information system of the SAGESSA Health Care Group (Spain)	• Analysed diagnostic and therapeutic medical procedures using temporal data from patient records, looked at six CPGs on chronic disease, synthesised two formalisms, micro- and macro-temporality and developed three algorithms for generalised time constraints	N/A	∘ Developed a temporal model capable of capturing chronic disease temporal knowledge from medical procedures • Adherence analysis detects CPG-compliant interventions but also deviations which can question CPG recommendations, e.g. if time between visits is too long	Case study with computer modelling. No time frame stated
Larson, USA, 2018 [16]	∘ Primary care providers in South Carolina who treated military service members and veterans in their clinics • 93 provided baseline info and 68 follow-up info (response rate 78.1%)	∘ Academic detailing (“provides in-office, interactive encounters with a clinical consultant (pharmacist) trained to assess the physician’s prescribing concerns and provide new information on recommended practices and tools”) • During this session, the pharmacist promoted three key messages about safer opioid prescribing	Within-group before-and-after intervention	∘ Pre- and post-self-report measures assessed by survey 4–38-week post intervention ∘ Used 2 pain specialists as champions to recruit physicians ∘ Feasibility, as assessed by number of new/reactivated prescription monitoring program (PMP) accounts • Effectiveness, as assessed by frequency of physician utilisation of PMP patient report data when prescribing opioids	Before-and-after study. May to November 2015
Moen, USA, 2019 [17]	• 28,179 Medicare beneficiaries who underwent implantable cardioverter defibrillator (ICD) therapy for primary prevention	∘ Review of registry data ∘ Clinical outcome of interest was guideline-consistent ICD implantation, calculated via the National CDR • Exposure variables: network measures of the ICD surgeon, referring hospital, hospital where the ICD surgery occurred	N/A	• Constructed physician and hospital networks for cardiovascular disease. Physicians were linked if they shared cardiovascular disease patients; these links were aggregated by hospital affiliation to construct a hospital network	Longitudinal, observational case study review of the association of guideline-consistent ICD placement 2007–2011
Mor, USA, 2000 [18]	• 1099 patients with stages 1 or 2 breast cancer treated at six Rhode Island hospitals	∘ Used diagnostic and treatment data from hospital tumour registries supplemented with patient self-reported adjuvant therapy information to assess rates of appropriate treatment in older women • Monitored performance and provided feedback to surgeons regarding treatment received by their breast cancer patients aged 60 years and older	Rates of appropriate treatment received before and after distribution of performance reports	∘ Presented a draft set of practice guidelines based on National Cancer Institute Consensus Conference recommendations to surgeons at meetings convened by the chiefs of surgery at six participating hospitals ∘ Collection of diagnostic and treatment data for all breast cancer cases diagnosed between 1992 and 1997 ∘ Tumour registrars conducted 5-min patient telephone interview (or mailed questionnaire) at ~4-month post-diagnosis. Survey provided uniform source of “real-time” data on radiation and hormonal therapy and chemotherapy • Summary data allowed surgeons to compare their own patients’ treatment with aggregated information on all women treated at the hospital	Before-and-after study. Nov 1, 1992, to Jan 31, 1997
O’Grady, USA, 2007 [19]	∘ Private medical oncology clinicians working in Fox Chase Cancer Center Partners (FCCCP — a community hospital/academic partnership consisting of 25 hospitals in the Delaware Valley)	∘ FCCC collaborates with community hospitals to translate NCCN guidelines to community-based private medical oncologists and monitor guideline compliance ∘ Concordance with multiple indicators assessed on 20 charts from each community practice ∘ A report for each FCCCP medical oncology practice summarises documentation, screening recommendations, new drug use and research trends in a particular disease site • Education and documentation tools are provided to physicians and oncology office nursing staff	N/A	∘ Phase I: education and dissemination of NCCN guidelines ∘ Phase II: selection of annual disease site and data management considerations ∘ Phase III: audit preparation, audit function and quality controls • Phase IV: audit report, implementation of best practice methodologies	Case study review of QI activities over the last 7 years. (since 1999)
Paxton, USA, 2012 [20]	∘ Medical centres in Kaiser Permanente (KP), the largest managed care organisation in the USA • All KP regions are eligible to participate in the registries, and most do	∘ KP developed and implemented several orthopaedic (total knee and hip replacement, hip fracture, shoulder arthroplasty) and cardiac registries (ICD, pacemaker, heart valve) ∘ These eight KP implant registries leverage the integrated healthcare system’s administrative databases and electronic health records system • Registry data collected undergo quality control and validation as well as statistical analysis	N/A	∘ Registries specifically developed to identify procedure incidence and implantable device utilisation, evaluate patient and device outcomes, identify patients at risk of poor clinical outcomes, identify and monitor devices in a recall/advisory situation, evaluate comparative effectiveness of devices, and serve as a foundation for quality improvement and research studies	Descriptive review of case studies of KP’s development of implant registries since 1999. Includes performance data
Rutledge, Australia, 2018 [21]	∘ 144 consecutive patients with newly diagnosed prostate cancer from the Hunter and Newcastle areas of NSW ∘ 75 pre-intervention groups; 69 post-intervention groups • Managed by one of nine urologists	∘ Multiphase intervention comprising review of current staging practices, education sessions led by clinical champions, deidentified review of urologist’s own practices and further education about clinical guidelines ∘ Primary outcome measure was use of CT and bone scan imaging, for staging purposes, in both pre-intervention and post-intervention study periods	Pre- and post-comparisons between two cohorts. Patients were also stratified into low-, intermediate- and high-risk groups within each cohort	∘ Audit of patient records (July 2014 to July 2015) ∘ Results presented to participating urologists by a clinical champion ∘ Urologists had focused education based on current guidelines, delivered as part of the local genitourinary oncology multidisciplinary team (MDT) • Clinical champion chaired an educational meeting where the evidence against the use of staging investigations for patients with low-risk, and in most patients with intermediate-risk, disease was presented ∘ Staging practices of deidentified urologists presented to the group, highlighting the overuse of staging investigations in men at low risk of metastatic disease • Education programme on current guidelines presented at MDT meetings	Before-and-after retrospective cohort study with before-and-after evaluation of intervention. July 2014–July 2016
Stark, Germany, 2013 [22]	∘ 975 post-myocardial infarction (MI) patients across Germany ∘ Response rate 90%	∘ Usual care was enrolment in a cardiac disease management program (CHD-DMPs) ∘ Intervention arm included the addition of secondary cardiovascular prevention guidelines (guideline care) • Guideline care was based on patient reports regarding medical advice (smoking, diet or exercise) and prescribed medications (statins and platelet aggregation inhibitors plus beta blockers or renin-angiotensin inhibitors)	Usual care	∘ A sample of patients with a previous MI who were enrolled in a CHD-DMP completed a questionnaire on their medical care (2006) ∘In CHD-DMP, regulations define appropriate therapies, follow-up and clinical parameters which are documented by physicians and submitted for evaluation ∘ Submitted data is used to provide individual feedback to physicians regarding their performance and to evaluate intermediate outcomes of CHD-DMP participants • Guideline-care group received additional advice on smoking, diet, exercise or medications	Prospective case study using survival analysis. Examined all-cause mortality, 2006–December 31, 2010
Viktrup, Denmark, 2004 [23]	∘ 243 general practitioners (GPs) in Frederiksborg County • Response rate of 132 (54%)	∘ Clinical guidelines for GP management of urinary incontinence (UI) were distributed in 2001 ∘ Government implemented a small reimbursement to GPs for use of a fluid intake/voiding diary in the assessment of UI in October 2001 • GPs were surveyed and information concerning monthly reimbursement for using a voiding diary, prescribed drugs (presumably used for treating UI), UI consultations in outpatient clinics and patient reimbursement for pads was collected	N/A	∘ GP survey on uptake of guidelines for management of UI • Included assessment of the impact of physician reimbursement	GP survey “distributed” in October 2001 (does not say by what means)

**Table 2:** Included CQR studies detailing population, intervention, comparator, outcomes and study design (PICOS)

Author, location, year	Population, setting, response rate	Intervention/evaluation	Comparator	Outcome: models/methods used	Study design and time frame
Ahern, Australia, 2020 [7]	Medical administrators, clinical risk and quality managers, medical heads of departments and principal investigators of clinical registries from the Alfred Hospital, Melbourne	The CQR initiative comprised three main components: (1) ascertaining AH participation in clinical registries, (2) engagement of clinicians in ongoing sharing of CQR outcomes and (3) piloting facilitation of CQR reports into AH clinical governance reporting	N/A	• Established a regular senior clinician Clinical Registry Interest Group as a forum for regular collaboration and discussion • Developed calendar schedule of CQR reports for monitoring by CGU • Incorporated clinical registry site data into existing unit-based safety and quality presentations • Developed guideline requiring units participating in CQRs to provide electronic copy of their reports to the CGU, with a summary of key report findings and action plan, if applicable, on a designated template • Created a CQR traffic-like style dashboard for each registry to allow benchmarked report data to be tracked and visualised over time	Case study of Alfred Health over 18 months (period not stated)
Algurén, Sweden, 2018 [24]	Clinicians (*n* = 185; 70% nurses, 26% physicians) via the NQRs’ email networks (121 Swedeheart users and 64 SwedeHF users)	Online survey to determine frequency of NQR use for (a) producing healthcare activity statistics, (b) comparing results between similar departments, (c) sharing results with colleagues, (d) identifying areas for quality improvement (QI), (e) surveilling the impact of QI efforts, (f) monitoring effects of implementation of new treatment methods, (g) doing research and (h) educating and informing healthcare professionals and patients	Clinician use of each registry; physician vs. nurse usage	• Survey of physicians and nurses about how they used data from two registries for nine purposes (see intervention) and how often they did so	A national online survey with users of two NQRs concerning cardiovascular healthcare, conducted between March and May 2016
Algurén, Sweden, 2019 [25]	Two quality improvement collaboratives in Sweden. Defined as a structured approach for improvement built on joint learning and with teams from multiple organisations	Final reports of two QICs—one on heart failure care with five teams and one on osteoarthritis care with seven teams, including detailed descriptions of improvement projects from each QIC’s team, were analysed and coded by 18 QIC characteristics and four team characteristics. Routinely collected goal variables from each team within the two registries analysed with univariate statistics	The other QIC. The two QICs differed greatly in design	• Described in detail and compared (a) the components of the respective QIC, (b) the characteristics and activities of each QIC team and (c) the longitudinal outcomes over 3 years after the launch of the QICs • Studied differences between QICs and between the teams and their activities, how they are linked and how they are inter-related with their longitudinal outcomes	Case study with a multiple-case embedded design, e.g. multiple units of analysis (2013–2016)
Cadilhac, Australia, 2017 [26]	Hospital clinicians involved in discharge care for stroke and patients admitted with acute stroke or transient ischaemic attack. Fifteen acute care public hospitals in Queensland, Australia	A four-stage, multifaceted organisational intervention including data reviews, education and facilitated action planning. Data on discharge care plan, antihypertensive medication and antiplatelet medication prescription (ischaemic events only) used to select hospitals. Primary measure: composite outcome. Secondary measures: individual adherence changes for each discharge process, sensitivity analyses. Performance outcomes compared 3-months pre-intervention, 3 months post-intervention and at 12 months (sustainability)	The other pilot hospital	• Delivery mechanisms: external facilitation, performance feedback with gap analysis, co-designed educational meeting using a local opinion leader, evidence from exemplar hospitals and structured action planning • Areas for improvement: inconsistent use of existing tools and systems, lack of pharmacist involvement, inconsistent procedural knowledge about discharge planning and suboptimal data recording in AuSCR and med records • Common strategies included providing example of a comprehensive discharge care plan for clinicians to refer to, using reminders (such as stickers in medical records) to facilitate prescription of medications and regular reviews of data at team meetings	A mixed-methods, controlled before-after observational study design
Cadilhac, Australia, 2019 [27]	19 of 23 eligible Qld hospitals (83%) and 23 others located elsewhere in Australia. Hospitals all contributed data to AuSCR and previous audits (data from 17,502 patients)	Baseline routine audit and feedback (control phase, 30 months), followed by two interventions: financial incentives (21 months) and the StrokeLink programme involving the addition of externally facilitated quality improvement workshops with action plan development (9 months). Post-intervention phase was 13 months	Historical controls and 23 other Australian hospitals (data from 20,484 patients)	∘ Financial incentives programme (2012) provided an incentive payment to increase access to stroke. Payments required a minimum proportion of data collected within AuSCR ∘ Enhanced StrokeLink programme: benchmarked feedback to clinicians on hospital performance and action plans to improve care ∘ From 2014, AuSCR clinical indicator and 90-day patient outcome data from the previous 12 months was provided • Other features: interactive discussion on actions to overcome local barriers and the provision of ongoing support via telephone or email	Multicentre, prospective, controlled, before-and-after, quality improvement study, 2010 to 2015
Eccleston, Australia, 2017 [28]	6720 consecutive patients undergoing percutaneous coronary interventions (PCIs) from 10 private hospitals across Australia (Qld, Vic, SA, WA)	Real-time benchmarking via a national clinical quality and outcomes register. GCOR-PCI prospectively collected clinical, procedural, medication and outcomes data from 6720 patients. The main outcome measure was compliance with guideline medications (statins, antiplatelet agents)	Benchmarking of treatment against trial evidence, international guidelines and practice	∘ Key performance outcomes benchmarked against the aggregated study cohort and international standards ∘ Benchmarked data reported to individual sites ∘ • Included measurement of quality of life at 30 days post-discharge and annually, using EQ-5D (not reported)	Before-and-after study comparing patient data for 2010 and 2014
Egholm, Denmark, 2019a [29]	175 staff in 30 hospital departments participating in the Danish Cardiac Rehabilitation Database Survey response rate was 58% (101/175)	A previously validated, Swedish questionnaire regarding use of data from CQRs was translated and emailed to frontline staff, mid-level managers and heads of departments (*n* = 175) in all 30 hospitals participating in the Danish Cardiac Rehabilitation Database	N/A	∘ Used the 50-item quality improvement while adopting quality register outcomes survey (QWAQ) ∘ Measured a range of aspects that may facilitate use of CQR data for QI work, including quality of clinical care, quality of registry data, organisational conditions for registry work and use of data for QI • Data were analysed descriptively and through multiple linear regression	Cross-sectional nationwide survey. Questionnaires emailed in May 2018
Egholm, Denmark, 2019b [30]	24 registry workers (12 each in England and Denmark). Sampled to maximise diversity. Mostly nurses, 23 women	Qualitative interviews with registry workers involved in collecting or entering data into the two registries	England and Denmark	• Content analysis of interviews produced one overarching theme “Struggling with practices” and five categories: the data entry process, registry quality, resources and management support, quality improvement and the wider healthcare context	Interview-based study conducted between Sept 2016 and April 2017
Eldh, Sweden, 2016 [31]	Managers, physicians and clinicians in all 72 Swedish stroke units. Response rate: 163/242 individuals (67.4%) from 70 units	A survey including 50 items on context, processes and the registry. Email reminders sent at 2, 3 and 4 weeks. A final reminder was sent after week 5 that included an opportunity to provide reasons for not partaking	N/A	∘ Survey comprised 50 questions organised in 7 sections: background information about the respondent, quality of care, data quality, organisational conditions, the respondent’s use of registry data, the stroke unit’s use of registry data, and perceived value of the registry • Data analysed descriptively and through multiple linear regression	Survey (distributed Sept. 2014 by email) is second phase in an exploratory sequential design
Granström, Sweden, 2018 [32]	All existing QRCs (quality registry centres) in Sweden	Document analysis and 25 semi-structured interviews with staff at 6 regional support centres, i.e. (QRCs). Data were analysed using conventional content analysis	Not stated	∘ Evaluation of how QRC staff understood their mission and role, perceived enablers and barriers for their work and the support strategies they used at each centre ∘ 25 semi-structured interviews were conducted twice with the same individuals (*n* = 13) ∘ Documents used to complement and contextualise the described strategies and to identify additional important information • Identified strategies mapped according to national or local focus and task- or process-oriented strategies	Multiple case study. Interviews in spring 2014 and 2015
Klaiman, USA, 2014 [33]	12 high-quality registries in the USA, Canada and Sweden, as determined by the research team and an expert panel	Defined characteristics of effectiveness and then studied examples of effective registries in cancer, cardiovascular care, maternity and joint replacement. A preliminary environmental scan identified examples of effective registry design and utilisation in terms of QI, value-based purchasing (VBP) and public reporting	N/A	∘ Based on results from the environmental scan, in-depth analyses of effective registries were conducted in 4 clinical areas ∘ An analytic framework called “positive defiance” used to understand factors that enable registries to be used successfully for quality monitoring and improvement • In-depth analyses were conducted with staff from 2 to 5 effective registries in each clinical area	Review of effective (positive deviant) registries. Included in-depth interviews with registry staff
Lipitz-Snyderman, USA, 2019 [34]	103 clinicians across Memorial Sloan Kettering Cancer Alliance invited to participate. 87 reported participation in a disease management team and were included in final analysis	Survey to physicians treating patients with cancer across the 3 Alliance member health systems, covering: awareness and perceived value of engagement opportunities through MSK Cancer Alliance, which engagement opportunities would they like and has clinical practice changed due to MSK membership. Plus open-ended comments and suggestions	N/A	∘ MSK Alliance provides opportunities for multidisciplinary clinicians to observe and present cases to MSK tumour boards, connect with MSK physicians for clinical input, attend clinical lectures, participate in MSK disease-specific retreats, provide local access to clinical trials for MSK patients and have MSK physicians participate in local meetings	Online survey. July–Sept. 2017
Løwer, Norway, 2013 [35]	Data from patients undergoing six surgical procedures in all hospitals in Norway	∘ Most Norwegian hospitals use computerised infection control modules (ICMs) to harvest data from the hospitals’ electronic patient admin systems and surgery scheduling systems • ICMs contain de-identified surgical data, module for manual input correction/override, automated generation of patient follow-up letters with bar codes, quality assurance routines and reports statistics generation for local use, quality assurance and submission to national level	Previous rates of surgical site infections	∘ All Norwegian hospitals participate in the surveillance system with data submitted to Norwegian Institute Public Health ▪ Infections monitored over a 3-month period each year ∘ 3 key features: (1) national and mandatory, (2) highly automated hospital data collection and (3) active post-discharge surveillance ∘ Database used to measure hospital participation, completeness of explanatory variables and post-discharge surveillance • - Data are collected before, during and 30 days after surgery (1 year for implants) and include explanatory and outcome variables; results were presented as proportions	Case study of a surveillance system for surgical site infections, using data from 2005 to 2009
Nag, Australia, 2019 [36]	159 cardiac surgeons from high-performing units invited. 24 (15%) surgeons responded to the initial survey; 20 completed responses were analysed	∘ 4 Victorian units contributing to the ANZCSTS database invited to participate ∘ Control and intervention groups each included one private and one public unit ∘ Cardiac surgeons surveyed to evaluate current feedback reports and assist in developing content of structured feedback • Intervention units received additional structured feedback	Routine practice. Unit performance also compared to national performance	∘ Online survey of 159 surgeons contributing data to ANZSCTS ∘ Two control units received current feedback reports, distributed quarterly to the head of unit and data manager ∘ Two intervention units received additional face-to-face structured feedback from an external surgeon at the unit’s quarterly multidisciplinary surgical review meeting ∘ Structured feedback customised to show unit-specific KPIs, highlighting areas of excellence and underperformance. • All participants completed short online study assessment survey	Before-and-after pilot study with online survey followed by targeted intervention in 2 of 4 cardiac units
Norman, Sweden, 2020 [37]	Use of SwedeHF (Swedish Heart Failure Registry) in one university hospital in one of the three biggest regions in Sweden	∘ Study of individuals’ decisions after programme focusing on increasing the use of NQRs • SwedeHF chosen because it is relatively new (2003) and less established. The effect of funding from the NQR programme was expected to be more obvious in SwedeHF than in a well-established, long-developed register	N/A	∘ Four contexts were identified: registration, use of output data, governance and improvement projects ∘ Used realist evaluation to identify contexts, mechanisms and outcomes • Explored different logics or “rules of the game” embedded in unconscious social norms that are part of work	Case study with 18 semi-structured interviews (2013–2015). Representatives from NQR contexts as well as the healthcare contexts were interviewed

### Identification of relevant information from the grey literature

KD undertook a simultaneous search of relevant grey literature using a list of suggested sources provided in the initial review template and expanded this list using a “snowballing” approach of checking relevant references and citations (see Additional file 2). The starting point for this search was the Australian Health Ministries Advisory Council (AHMAC) and ACSQHC strategy document [1].

### Analytic approach

Data were extracted from included studies and tabulated and synthesised using thematic analysis methods. Findings were grouped and summarised using an inductive and iterative approach. A range of overarching themes were generated [38], highlighting strategies used to support the effective use of clinical practice guidelines and clinical quality registry data to inform and improve health service delivery.

### Quality assessment

Quality assessment was undertaken using checklists relevant to each included articles’ study design. Seventeen studies were assessed using the Mixed Methods Appraisal Tool (MMAT), a validated critical appraisal tool relevant for qualitative, quantitative RCTs, quantitative non-randomised, quantitative descriptive, and mixed methods study designs [39]. Each study type was assessed using five questions, with three possible answers: yes, no and cannot tell (Additional file 3). This tool does not recommend calculating an overall score or excluding studies with low methodological quality [39]. In total, 9 studies met all five criteria [17, 21, 22, 25, 30–32, 37], two met four [26, 29], one met three [24], four met two [16, 18, 23, 34] and one met none [36].

Four studies [7, 13, 19, 27] were assessed using the Quality Improvement Minimum Quality Criteria Set (QI-MQCS, Version 1.0) tool, a valid and reliable critical appraisal instrument applicable to healthcare quality improvement (QI) interventions [40]. It comprises 16 criteria, with responses of either “met” or “not met” [40] (see Additional file 4). Of these four studies, the overall quality was very high: one met 15 of the 16 criteria [19], two met 14 [13, 27] and one met 13 of the 16 criteria [7].

Four articles could not be assessed for quality with existing tools as they documented processes and procedures of effective registries [20, 28, 33, 35]. A further publication, which described decision support mechanisms, could not be assessed for quality as it required specialist IT evaluation [15].

## Results

Figure 1 outlines the selection process. One-hundred and fifty-one CPG-related abstracts and 191 CQR-related abstracts, excluding duplicates, were identified. Of these, 21 CPG and 27 CQR abstracts met eligibility criteria and underwent full-text review (inter-rater reliability scores of 83.5% were attained for the guideline abstracts and 79.2% for the CQR abstracts). A second round of eligibility review of full-text articles, their references and citations resulted in 100% agreement between KD and CF (11 CPG articles and 15 CQR articles; *n* = 26).

### Clinical practice guidelines

The 11 CPG articles included only one randomised controlled trial (RCT) [14], four before-and-after studies [13, 16, 18, 21], four case studies [15, 17, 19, 22], one descriptive study [20] and one survey [23]. The lack of RCT design in ten of the 11 studies meant causation could not be determined, and generalisability of their findings to other settings, conditions or health systems was limited. The therapeutic areas covered included the following: hospital-acquired infections following caesarean section, opioid prescription rates, medical oncology practice, breast cancer, prostate cancer, urinary incontinence, implantable orthopaedic and cardiac devices and atrial fibrillation.

Eight of the 11 CPG articles mentioned registry data [13, 16–20, 22, 23] mostly in terms of using that data to assess the impact of the intervention. Five studies were conducted in the United States of America (USA) [16–20] with one each from Sweden [13], the Netherlands [14], Slovenia/Spain [15], Australia [21], Germany [22] and Denmark [23]. Table 1 shows the PICOS data, while study findings are summarised in Additional file 5.

### Clinical quality registries

The 15 CQR articles included the following: four before-and-after studies [26–28, 36]; four survey studies [24, 29, 31, 34]; four case studies [7, 25, 32, 35]; and three interview studies [30, 33, 37]. Again, the lack of rigorous study design limited the confidence in findings from these studies and their generalisability. The therapeutic areas included the following: six cardiac, three stroke, one cancer, one surgical site infections and four were non-specific. Five articles reported registry processes not restricted to a specific health condition. Five studies each were from Sweden [24, 25, 31, 32, 37] and Australia [7, 13, 26, 27, 36], two from USA [33, 34], one joint study from Denmark/England [30] and one each from Denmark [29] and Norway [35]. PICOS information is available in Table 2, and study findings are summarised in Additional file 6.

### Findings

This review identified five strategies that strengthen adherence to CPGs and utilise CQRs for quality assurance and benchmarking to facilitate best practice health service delivery.

#### Strategy 1: Providing high-quality feedback on data with transparency of findings

One study evaluated the impact of surgeon-specific performance reports on adherence to treatment guidelines for older women with breast cancer [18]. The authors concluded surgeon feedback “was insufficient to alter established practices”, and guideline adherence data may need to be used as a standard quality indicator of clinician practice to encourage improved adherence [18]. A review of the Australian and New Zealand Society of Cardiac and Thoracic Surgeons (ANZSCTS) database reported structured feedback did not significantly improve communication or QI as a result of high unit performance at baseline, low surgeon participation, scheduling challenges for structured feedback and limited dissemination to the wider surgical team [36]. In contrast, Paxton et al. [20] report that, back in 2012, eight national Kaiser Permanente (KP) implant registries, covering orthopaedic and cardiac devices, were electronically capturing information from administrative and claim databases to supplement the registry databases. All KP registries include patient demographic data (age, sex, body mass index, ethnicity), diagnosis, devices used, enrolment history and surgical outcomes including revision procedures, surgical site infections, other complications and death. This integrated data system allowed KP to communicate quality improvement and benchmarking information throughout their networks. For example, registry findings were communicated to widespread audiences of surgeons, administrators and clinical staff through a range of fora including “chiefs of service and administrator meetings, the internal website, individualised physician practice profiles, site visits, newsletters, e-mails, and presentations at regional or national conferences” [20]. Furthermore, reports could be generated and communicated at the level of medical centres and even individual surgeons. Centre-specific reports were made available on the KP internal website, and surgeons could request their own performance reports via “secure communication” routes. Such targeted reporting and dynamic feedback provide the opportunity for benchmarking across sites, regions and nationally and “yields measurable objective improvements in care”, as evidenced by an overall decrease in the burden of total hip replacement revision since the implementation of the KP Total Joint Replacement Registry in 2001 [20]. The American Society of Thoracic Surgeon’s Adult Cardiac Surgery Registry goes further, providing information for public reports that rank cardiac surgery groups [33].

#### Strategy 2: Planning for sustainability of successful interventions

Sustainability of QI is another important consideration. In a study of post-discharge stroke care, the authors noted the importance of measuring “long-term sustainability impact” [26]. A comparison of two cardiovascular quality registries in Sweden found activities focusing on adherence to standard care programmes and on increased follow-up of patients seemed to lead to more long-lasting outcome improvements [24]. Similarly, a Swedish study argued that surveillance of infectious conditions should be continuous, based on findings that post-caesarean surgical site infection rates decreased from 17.4 to 2.6% within the first year of the intervention but increased to 6.4% in the second year. Reinforcement of the intervention measures resulted in a further reduction of the infection rate to 1.1% [13]. An Australian study of prostate cancer imaging reported a focused, clinician-centred education programme led to improved guideline adherence at a regional level [21]. However, this effect did not persist once the intervention was complete, raising the importance of monitoring outcomes and reinstating successful interventions as needed [21].

A direct mechanism for sustainable CQRs is the legal mandate to report specific disease conditions, as noted in the USA in relation to cancer reporting [33] and in Sweden [13] and Norway [35] with respect to infected surgical sites. In addition, the role of financial incentives improves sustainability in non-mandatory registries. For example, in the USA, cardiologists are not legally required to submit patient care information to cardiovascular registries; however, registry participation is increasingly becoming a prerequisite of eligibility for bonus programmes, preferred provider network status and reimbursement [33]. These payer incentives provide a financial lever for on-going registry participation which is now relatively high among cardiologists and cardiac surgeons [33].

Guaranteed funding is another major component of sustainability. In the USA, cardiovascular registries have found a variety of ways to maintain financial stability, including subsidies from pharmaceutical and device manufacturers, dues paid by participating providers and revenues from the sale of registry data and from grants/contracts for studies derived from those data [33]. Funding models are evolving, and financial incentives are playing a growing role. Participation in cardiovascular registries is increasingly required as a condition of eligibility for bonus programmes, preferred provider network status, and reimbursement [33]. The Society of Thoracic Surgeons (STS) registries are funded through STS member dues, data sales and research grants rather than industry funds [33]. The US federal government funds state cancer registries, and many of these that make data available for researchers receive grant and/or partnership funds from academic institutions [19, 23].

In Sweden, registry governance and funding are derived from its 21 regional counties and include financial levers, such as incentive payments to healthcare providers, to participate [25]. While the introduction of pay for performance has led to an increased focus on NQRs in Sweden, it has also led to increasing competition between registers [37]. A Danish study on adoption of urinary incontinence guidelines found 35% of GPs received reimbursement during the first year after the introduction of payment for using a voiding diary [23]. Another Danish study reported that nonmonetary incentives, such as improving patient care and raising acknowledgement for cardiac rehabilitation, emerged as less tangible but strong incentives [29]. In contrast, participation in Australian CQRs “is largely an unfunded activity at the health service level, with neither funder nor health service funding being allocated to collection or review of these data” [7]. An Australian study of QI interventions in stroke included an incentive payment to increase access to stroke care, contingent on a clinician’s minimum proportion of data collected within the Australian Stroke Clinical Registry. The specific impact of the financial incentive alone was not clear however, because other elements were included in the intervention programme [27].

While funding is crucial to provide long-term CQR viability, additional spending does not necessarily lead to improved outcomes. When a Swedish heart failure registry and osteoarthritis registry were compared, there was no difference in the number of sustained improvements of the chosen goal indicators despite substantial additional funding to the heart registry [25]. Therefore, national standards, mandated reporting and public financing as evidenced in the US state cancer registries may be a more effective base for sustainable and effective interventions.

#### Strategy 3: Collecting, monitoring and reporting data on adherence to CPGs

One way to improve the quality of care is by providing evidence-based care aligned with clinical guideline recommendations [32]. Improvement in adherence to guidelines has been achieved by the Swedish government in acute myocardial infarction and diabetes care through support of more than 100 national quality registries and utilising quality improvement collaboratives (QICs) [25]. A different example shows improved adherence to opioid-prescribing guidelines has been achieved in the USA through a single visit with a trained academic detailer [16]. Unfortunately, no data was available on longer-term adherence.

Models of care associated with improved guideline adherence includes the Fox Chase Cancer Center partners community hospital/academic partnership model, which uses a tumour registry for disease-specific data and undertakes regular audits of participating hospitals for adherence to the National Comprehensive Cancer Network Clinical Practice Guidelines in Oncology [19]. The Kaiser Permanente group of registries verify adherence to national practice guidelines, such as indications for implantable cardioverter defibrillators as established by the American Academy of Cardiology [20]. A study of the National Cardiovascular Data Registry (NCDR) suggested regionalisation of specialised services, in which peripheral hospitals refer to regional centres (“hub and spoke”) model, may be associated with improved guideline adherence [17]. A German study reported patients enrolled in cardiac disease management programmes (CHD-DMPs) were more likely to receive guideline care, although CHD-DMP participation itself did not significantly improve survival [22]. Other studies have found surgeon-related factors, such as mapping surgeon networks [17] and individual surgeon characteristics [18], did not influence guideline adherence.

Two studies on guideline adherence stressed the importance of electronic data systems for decision support. The development of timed medical decision support systems can be used to manage new chronic patients, to audit the clinical actions performed in healthcare centres and to analyse the adherence of clinical actions to the standard procedures described in CPGs [15]. A Dutch RCT reported guideline recommendations for treatment of atrial fibrillation were more comprehensively implemented in the intervention group (nurse-led care) than usual care [14]. The authors suggested this was most likely due to the use of an electronic patient record with incorporated dedicated decision support software based on the guidelines that downsize complexity and improve adherence to recommendations. Another possibility the authors acknowledged is that a multidisciplinary approach in which cardiologists and nurses work closely together and are required to justify protocol deviations may preclude treatment decisions that do not comply with guidelines so facilitating QI [14].

#### Strategy 4: Forming productive partnerships

Several National Cancer Institute (NCI)-designated Comprehensive Cancer Centers in the USA have established academic and clinical partnership models, including the Duke Cancer Network, MD Anderson Cancer Network, the Memorial Sloan Kettering (MSK) Cancer Centre Alliance [34] and Fox Chase Cancer Center Partners Program [19]. These programmes typically provide community hospital partners with access to consultative services, treatment protocols, research and clinical trials. In return, the academic centres obtain enhanced accrual to and more timely completion of clinical trials and the inclusion of more diverse populations in these studies [34]. These partnerships with community health systems facilitate economies of scale, with shifts to population health and value-based reimbursement models, clinical standardisation and increased consumer engagement [34]. MSK Cancer Alliance taps into routine internal data to benchmark practice for each MSK-determined standard of care. Using data from each institution’s tumour registry and billing and medical records, MSK identifies practice variation within the system and uses this information to inform strategies to address gaps [34].

Productive partnerships between professional associations that address a specific health condition are illustrated by the leading cardiovascular societies in the USA. The American College of Cardiology, the STS and the American Heart Association share several collaborative registries in addition to individual registries [33]. These registries use data integrators, establish partnerships between professional societies in registry design and implementation, implement creative mechanisms for registry financing, provide rapid feedback to providers and use consumer-oriented products to share outcome data [33]. The PINNACLE registry (for outpatient cardiology quality improvement) can directly report back measures to the Centres for Medicare and Medicaid Services and to providers at the individual physician or group level and benchmark performance measures to national averages [33].

#### Strategy 5: Engaging with the whole CQR team

Several studies have reported the low levels and/or infrequent use of registry data to inform clinical practice or continuous QI activities [8, 20, 29–33]. Where registries often involve siloed registry staff, QI initiatives usually involve frontline staff that have varying knowledge and experience in interpreting registry data and incorporating it into multidisciplinary clinical decision-making [8, 32]. Several solutions have been suggested to engage with the QR team. First, the quality measurement efforts need to be adapted to the requirements of end users. Linking registration of quality indicators to use for QI activities increases their routine use, thus making them meaningful tools for clinicians [8]. Second, simplifying the process by tying data entry to existing routines, such as same day entry, and the use of a more user-friendly technology interface, would facilitate registry use [29]. A third potential solution is to focus on one or two key elements that can help registries be better leveraged for improving healthcare performance [33]. For example, the KP implant registries leveraged the integrated healthcare system’s administrative databases and comprehensive health electronic records [20]. KP used risk calculators, based on multivariable analyses of registry data, which allowed patients and surgeons to make clinical decisions at the point of care. In addition, they were able to use administrative data to conduct performance evaluations comparing similar implants with patient and surgical characteristics [20].

A fourth solution centres on a supportive organisational culture. Findings from interviews with registry workers in England and Denmark noted that management support in the data collection and entry phase was crucial. Their findings showed that getting high-quality data into a registry requires a dedicated, sustained effort that involves not only staff but also all stakeholders [29]. Developing a culture of data reporting may facilitate CQR implementation, along with highly engaged teams or individuals, who act as champions for change [29]. The success of the Swedish Riksstroke registry data for QI was strongly related to the buy-in and interest from managers, physicians and nurses in all 72 Swedish stroke units, and that QI initiatives were facilitated by joint ventures between health professionals, managers and policy-makers [31]. Similarly, in Australia, CQR clinician champions were critical in highlighting the benefits of sharing CQR results within the health service [21]. Clinical champions have a role to counter medical doctors’ influence when it presents as a barrier to new initiatives reaching clinical care [37].

The final solution highlights the importance of ongoing communication. In a large metropolitan hospital in Australia involved in 69 data registries, a range of communication strategies were tested to maintain engagement of clinicians in sharing CQR outcomes. Strategies included the establishment of a registry interest group, a calendar of clinical quality registry reports and a reporting guideline, template and dashboard. The reporting tool and dashboard have shown success at the pilot stage [7]. Communication is required not only within organisations but also between different levels of healthcare organisations responsible for delivering improved patient care. Table 3 highlights the complexities of healthcare delivery when different sections of the health system have different roles and responsibilities.

**Table 3:** Examples of strategy components and levels of responsibility

Strategy	Clinician level	Health services level	Health systems level
1. Feedback and transparency	Feedback from a trusted source, dissemination of findings to all stakeholders	Embedding CQR reports into health services reporting frameworks, groups of disease-specific registries use consumer-oriented products to share outcome data, direct reporting to government agencies	Benchmarking against best practice, public reporting of data
2. Intervention sustainability	Embedding CQR activities into routine clinical care, adherence to standard care programmes, more regular follow-up of patients, monitoring outcomes and reinstating successful interventions as needed	Integrated administrative and registry databases, individual registry funding through subsidies from pharmaceutical and device manufacturers, dues paid by participating providers and revenues from the sale of registry data and from grants/contracts for studies derived from those data	Guaranteed funding, mandatory participation in CQRs, legal mandate to report specific disease conditions, financial incentives for non-mandatory registries, e.g. payments for clinician participation, registry participation a condition of eligibility for bonus programmes, preferred provider network status and reimbursement
3. Clinical practice guideline adherence	Ongoing monitoring of trusted data, registry verification of adherence to national practice guidelines, patients enrolled in disease-specific management programmes, electronic data systems for decision support, multidisciplinary teams required to justify protocol deviations	Community hospital and academic partnership models identify practice variation to inform strategies to address gaps, regionalisation of specialised services (“hub-and-spoke” model), nonmonetary incentives such as improving patient care	Quality improvement collaboratives
4. Productive partnerships	High levels of participation in registry input	Grant and/or partnership funds from academic institutions, partnerships between professional societies in registry design and implementation	Creation of broad clinician communities of practice, sharing of best practice evidence
5. Whole-of-team approach	Clinician champions; buy-in and interest from managers, physicians and nurses; communication strategies, e.g. registry interest group, calendar of CQR reports, reporting guidelines, templates and dashboard; tying data entry to existing routines; user-friendly technology interface; linking registry quality indicators with QI activities	Local management support for the initiatives, careful registry design to maximise usability and usefulness, use of integrated data for research and clinical practice, e.g. risk calculators	Sustainable funding and support for CQRs, including registry design and development of innovative research methods; improved coordination of national registries and greater dissemination of information on how successful registries operate

## Discussion

This comprehensive systematic review of CPGs and CQRs has identified five important strategies to facilitate the translation of research evidence and data into practice, across a range of health conditions and countries. This set of strategies, applied in context-relevant combinations, is likely to be the most successful tools for informing health services delivery options and facilitating those options that have the best chance of improving health outcomes across different health settings, conditions and systems.

The defining feature of a CQR is the feedback loop to clinicians which transforms it from a data repository into the change management component of a LHS [1]. Transparent, high-quality and timely feedback allows individual clinicians to assess how their patient outcomes compare with others and to identify gaps in their own practice that need improvement (strategy 1). This view is supported by a Cochrane review of audit and feedback (A&F) mechanisms that reported feedback worked best with under-performing health professionals, and when it was delivered by a supervisor or colleague, was provided more than once, verbally and in writing and included clear targets and an action plan [41, 42]. These mechanisms may also work to improve the use and usability of registry data. For example, the open reporting systems that form the basis of the Kaiser Permanente registry groups have been successful in providing accessible and useful data to administrators and clinicians that has been used for audit and feedback as well as QI and benchmarking [20].

Sustainability strategies (strategy 2) are echoed in the 2017 Cochrane Effective Practice and Organisation of Care (EPOC) systematic review guidelines which specify a minimum of three data collection time points both before and after an intervention to be eligible for inclusion in a Cochrane EPOC systematic review [43].

Adherence to CPGs (strategy 3) is one of two major ways of benchmarking health services around adherence to best evidence practice. Monitoring adherence with evidence-based CPGs is the primary purpose of some registries in the United States of America (USA). There are examples where even this basic use of registry data appears to have increased use of evidence-based therapy, more efficient patient care and shorter hospital stays [44]. A second method of benchmarking is through comparison of current outcomes against best clinical practice and through trialling QI measures that have been successfully implemented in high-performing clinical settings (strategy 4).

Effective CQR outputs, capable of identifying gaps in services and providing reliable data for benchmarking, require sustained effort by the whole team including those involved in CQR design, management, data collection, data entry and data usage (strategy 5). LHSs aim to simplify the link between knowledge generation and evidence implementation and represent the perfect environment for maximising potential benefits from CQRs. In practice, however, there are few examples of where these systems work well [45].

Overall, the quality of included studies was high. Twenty of the 26 publications were able to be assessed using quality criteria; of these, 15 could be considered high quality (eleven scored 4 or 5 out of 5 on one measure (Additional file 3), and four scored 13 or higher out of 16 on another measure (Additional file 4). However, there was only one RCT in this review; the other study designs (before-and-after, case study, interview and survey designs) were not capable of determining causation of any outcomes. If the aim of a study is to determine whether a specific intervention did or did not lead to quality improvement in patient care, then an RCT design is required with individual components randomised separately to avoid confounding of effects.

More efficient usage of CPG evidence and CQR data would be facilitated by improved coordination of national registries and greater dissemination of information on how successful registries operate. For example, the authors and AHRA Working Group are aware of several high-impact registries that were not represented in the peer-reviewed literature. While some information about these registries is available through their respective websites (see Additional file 2), strategies they employ to inform healthcare delivery are not available. An earlier systematic review argued that evidence of CQR impact does exist, but is not available for review because the empiric studies have not been published [11]. This is a general limitation of reviews in this field.

Greater coordination of registries on a national basis would enable newer CQRs to benefit from the methods and models used by their more experienced counterparts. The Australian Commission on Safety and Quality in Health Care is well-placed to oversee the further development of CQRs and is currently working on a revised framework and the development of quality standards for CQR governance, reporting and technical infrastructure arrangements, to minimise duplication and maximise the use of health data for the community [46].

Innovative methodologies such as implementation laboratories, which encourage the partitioning of major research projects into smaller, more manageable parcels and the sharing of knowledge between groups working in the same area, are one example of a promising approach [45]. Rather than a plethora of poorly designed research projects trying to answer difficult questions about what works best, fewer research groups could focus on a smaller part of the puzzle, with a targeted approach using rigorous methodology [45].

There are several other areas of potentially useful research in this field. Quantitative surveys and qualitative studies that explore the reasons for non-publication of successful examples of best practice are needed to determine the extent of under-reporting and propose solutions. Such research could explore the potential role of positive or negative incentives to promote better coordination and publication of research and consider a greater role for patient views on what works best for the service users through the incorporation of patient-reported outcome and experience measures. The use of hybrid study designs that aim to simultaneously consider both the intervention and the implementation strategies used to promote uptake [47, 48] may also be useful.

### Strengths/limitations

This review was rigorously conducted and satisfied the PRISMA-S checklist criteria [49] (see Additional file 7). It covered publications reporting guideline and registry use in 10 countries over a 20-year time period and was not limited to specific health conditions. Analysis of findings was descriptive, as determined by the range of study designs. Despite a thorough search strategy, some relevant publications may have been missed. Reference and citation checking, along with consultation with experts in the field, likely reduced this possibility. While individual primary studies in this review contained variable degrees of selection, confounding and reporting bias, the quality assessment of each study was tailored to the different study designs to account for this.

## Conclusion

This extensive review of CPG and CQR studies aimed at improving health service delivery identified a set of five key strategies that have been used to facilitate the translation of research evidence/data into practice across a range of health conditions and countries. The use of these strategies, in different combinations according to their potential utility to address issues in different contexts, may provide a helpful framework for considering options demonstrated to have worked in similar contexts. Greater coordination and knowledge sharing, in conjunction with higher-quality research methodology, are also required to improve evidence uptake, health service delivery and patient outcomes.

## Supplementary Information

**Additional file 1.** Literature search strategies

**Additional file 2.** Grey literature

**Additional file 3.** Critical appraisal using MMAT

**Additional file 4.** Critical appraisal using QI-MCQS

**Additional file 5.** CPG results [Clinical Practice Guideline results]

**Additional file 6.** CQR results [Clinical Quality Registry results]

**Additional file 7.** PRISMA-S checklist

## Data Availability

All data generated or analysed during this study are included in this published article (and its supplementary information files).
