# Supplementary material for: Which strategies support the effective use of clinical practice guidelines and clinical quality registry data to inform health service delivery? A systematic review
Source: Syst Rev. 2022 Nov 9;11:237. doi: 10.1186/s13643-022-02104-1 (PMC9644489; doi:10.1186/s13643-022-02104-1)

**Literature search strategies**A systematic literature search was conducted between 3-20 March 2020 using two separate strategies.

**Search 1.**

Five databases were searched for relevant articles that contained *BOTH* “Guidelines” *AND* “Registries” as keywords, limited to the timeframe between 2000 and 2020.

**Search terms used were:**
1. Guideline Adherence/

2. ((register* or registri* or protocol* or guideline*) adj4 (compliance or comply or integrat* or adhering or adherence)).mp.

3. 1 or 2

4. exp "Delivery of Health Care"/

5. (health care adj4 (provision or delivery or model* or method* or system*)).tw.

6. 4 or 5

7. ((register* or registr*) adj4 (data or information)).tw.

8. 3 and 6 and 7

9. limit 8 to (english language and humans and yr="2000 -Current")

**Results:**

- Medline – 114 articles (Ovid)
- Embase – 27 articles (Ovid)
- CINAHL – 3 articles (University of Sydney Library Ebsco Databases)
- Cochrane Central – 3 articles (via Ovid)
- Cochrane Database of Systematic Reviews 5 – articles (via Ovid)

**Total:**
152 abstracts. When exclusion criteria were applied and duplicates removed, this left 101 abstracts for review (as agreed by KD and CF).

**Search 2.**

Four databases were searched in the second literature search conducted on 30 March 2020 to capture relevant articles that contained *EITHER* the term “guidelines” *OR* the term “registries”, again limited to the timeframe 2000-2020. The Cochrane Database of Systematic Reviews was not searched again because this was only searched initially to identify other systematic reviews performed in this field – systematic reviews were excluded from the current systematic review.

**Search terms used were:**

1. (clinical quality adj4 (registr* or register*)).mp.

**Results:**

- Medline – 106 articles
- Embase – 177 articles
- CINAHL – 199 articles
- Cochrane Central – 12 articles

**Total:** 494 abstracts. When exclusion criteria were applied and duplicates removed, this left 130 abstracts for review (as agreed by KD and CF).

**Excerpts from actual searches, reproduced as per PRISMA S checklist**

**Medline search 1**

Results: 114

| **#** | **Search Statement** | **Results** |
| --- | --- | --- |
| 1 | Guideline Adherence/ | 31342 |
| 2 | ((register* or registri* or protocol* or guideline*) adj4 (compliance or comply or integrat* or adhering or adherence)).mp. | 45189 |
| 3 | 1 or 2 | 45189 |
| 4 | exp "Delivery of Health Care"/ | 1055627 |
| 5 | (health care adj4 (provision or delivery or model* or method* or system*)).tw. | 62513 |
| 6 | 4 or 5 | 1088694 |
| 7 | ((register* or registr*) adj4 (data or information)).tw. | 31722 |
| 8 | 3 and 6 and 7 | 123 |
| 9 | limit 8 to (english language and humans and yr="2000 -Current") | 114 |

**Embase search 1**

Results: 27

| Embase Classic+Embase <1947 to 2020 March 05> | | | |
| --- | --- | --- | --- |
| **#** | **Search Statement** | **Results** | **Annotation** |
| 1 | protocol compliance/ | 12632 |  |
| 2 | ((register* or registri* or protocol* or guideline*) adj4 (compliance or comply or integrat* or adhere* or adhering or adherence)).mp. | 44199 |  |
| 3 | 1 or 2 | 44199 |  |
| 4 | exp health care delivery/ | 3257168 |  |
| 5 | (health care adj4 (provision or delivery or model* or method* or system*)).tw. | 85375 |  |
| 6 | 4 or 5 | 3306127 |  |
| 7 | Benchmarking/ | 4672 |  |
| 8 | [benchmark*.tw.](http://scanmail.trustwave.com/?c=13000&d=-LHm3mHh-itxA4FsjCq-JXZBd9az4TBPSg6BLS2hZw&u=http%3a%2f%2fbenchmark%2a%2etw) | 47651 |  |
| 9 | 7 or 8 | 48735 |  |
| 10 | register/ | 115012 |  |
| 11 | (register* or registr*).mp. | 580462 |  |
| 12 | 10 or 11 | 580462 |  |
| 13 | (data or information).mp. | 6608230 |  |
| 14 | 12 and 13 | 301170 |  |
| 15 | 3 and 6 and 9 and 12 and 14 | 27 |  |

**Cinahl Search 1**

Results: 3

| **Search ID#** | **Search Terms** | **Search Options** | **Last Run Via** | **Results** |
| --- | --- | --- | --- | --- |
| S10 | S3 AND S6 AND S7 AND S8 AND S9 | Expanders - Apply equivalent subjects  Search modes - Boolean/Phrase | Interface - EBSCOhost Research Databases  Search Screen - Basic Search  Database - CINAHL Complete | 3 |
| S9 | (MH "Benchmarking") | Expanders - Apply equivalent subjects  Search modes - Boolean/Phrase | Interface - EBSCOhost Research Databases  Search Screen - Basic Search  Database - CINAHL Complete | 6,820 |
| S8 | data | Expanders - Apply equivalent subjects  Search modes - Boolean/Phrase | Interface - EBSCOhost Research Databases  Search Screen - Basic Search  Database - CINAHL Complete | 1,131,172 |
| S7 | (register* or registr*) | Expanders - Apply equivalent subjects  Search modes - Boolean/Phrase | Interface - EBSCOhost Research Databases  Search Screen - Advanced Search  Database - CINAHL Complete | 174,065 |
| S6 | S4 OR S5 | Expanders - Apply equivalent subjects  Search modes - Boolean/Phrase | Interface - EBSCOhost Research Databases  Search Screen - Advanced Search  Database - CINAHL Complete | 351,634 |
| S5 | (health care) N4 (provision or delivery or model* or method* or system*) | Expanders - Apply equivalent subjects  Search modes - Boolean/Phrase | Interface - EBSCOhost Research Databases  Search Screen - Advanced Search  Database - CINAHL Complete | 115,358 |
| S4 | (MH "Health Care Delivery+") | Expanders - Apply equivalent subjects  Search modes - Boolean/Phrase | Interface - EBSCOhost Research Databases  Search Screen - Advanced Search  Database - CINAHL Complete | 309,413 |
| S3 | S1 OR S2 | Expanders - Apply equivalent subjects  Search modes - Boolean/Phrase | Interface - EBSCOhost Research Databases  Search Screen - Advanced Search  Database - CINAHL Complete | 20,843 |
| S2 | (register* or registri* or protocol* or guideline*) N4 (compliance or comply or integrat* or adhering or adherence) | Expanders - Apply equivalent subjects  Search modes - Boolean/Phrase | Interface - EBSCOhost Research Databases  Search Screen - Advanced Search  Database - CINAHL Complete | 20,843 |
| S1 | (MH "Guideline Adherence") | Expanders - Apply equivalent subjects  Search modes - Boolean/Phrase | Interface - EBSCOhost Research Databases  Search Screen - Advanced Search  Database - CINAHL Complete | 13,876 |

**Cochrane Central 1**

Results: 3

| EBM Reviews - Cochrane Central Register of Controlled Trials <February 2020> | | | |
| --- | --- | --- | --- |
| **#** | **Search Statement** | **Results** | **Annotation** |
| 1 | Guideline Adherence/ | 1040 |  |
| 2 | ((register* or registri* or protocol* or guideline*) adj4 (compliance or comply or integrat* or adhering or adherence)).mp. | 8296 |  |
| 3 | 1 or 2 | 8296 |  |
| 4 | exp "Delivery of Health Care"/ | 42130 |  |
| 5 | (health care adj4 (provision or delivery or model* or method* or system*)).tw. | 4201 |  |
| 6 | 4 or 5 | 45838 |  |
| 7 | exp Registries/ | 963 |  |
| 8 | (register* or registr*).tw. | 92837 |  |
| 9 | 7 or 8 | 93065 |  |
| 10 | [data.tw.](http://scanmail.trustwave.com/?c=13000&d=4pDn3qGb4-DFLjEzxNgB4Wfgrq92XMcYd0y9BljV7Q&u=http%3a%2f%2fdata%2etw) | 273344 |  |
| 11 | Benchmarking/ | 101 |  |
| 12 | [benchmark*.tw.](http://scanmail.trustwave.com/?c=13000&d=4pDn3qGb4-DFLjEzxNgB4Wfgrq92XMcYd027UFWHvA&u=http%3a%2f%2fbenchmark%2a%2etw) | 1310 |  |
| 13 | 11 or 12 | 1353 |  |
| 14 | 3 and 6 and 9 and 10 and 13 | 3 |  |

**Cochrane Database of Systematic Reviews 1**

Results: 5

| EBM Reviews - Cochrane Database of Systematic Reviews <2005 to March 4, 2020> | | | |
| --- | --- | --- | --- |
| **#** | **Search Statement** | **Results** | **Annotation** |
| 1 | ((register* or registri* or protocol* or guideline*) adj4 (compliance or comply or integrat* or adhering or adherence)).mp. | 516 |  |
| 2 | (health care adj4 (provision or delivery or model* or method* or system*)).tw. | 315 |  |
| 3 | (register* or registr*).tw. | 9386 |  |
| 4 | [data.tw.](http://scanmail.trustwave.com/?c=13000&d=kZHn3vVzRBcgv6PCWvns5lw33g0UjptLLovChO_ojg&u=http%3a%2f%2fdata%2etw) | 10482 |  |
| 5 | [benchmark*.tw.](http://scanmail.trustwave.com/?c=13000&d=kZHn3vVzRBcgv6PCWvns5lw33g0UjptLLorE0uK63w&u=http%3a%2f%2fbenchmark%2a%2etw) | 130 |  |
| 6 | 1 and 2 and 3 and 4 and 5 | 5 |  |

**Medline search 2**

Results: 106

| Ovid MEDLINE(R) ALL <1946 to March 26, 2020> | | | |
| --- | --- | --- | --- |
| **#** | **Search Statement** | **Results** | **Annotation** |
| 1 | (clinical quality adj4 (register* or registr*)).mp. | 106 |  |

**Embase search 2**

Results: 177

| Embase | | | |
| --- | --- | --- | --- |
| **#** | **Search Statement** | **Results** | **Annotation** |
| 1 | (clinical quality adj4 (register* or registr*)).mp. | 177 |  |

**Cochrane Central search 2**

Results: 12

| EBM Reviews - Cochrane Central Register of Controlled Trials <February 2020> | | | |
| --- | --- | --- | --- |
| **#** | **Search Statement** | **Results** | **Annotation** |
| 1 | (clinical quality adj4 (register* or registr*)).mp. | 12 |  |

**CINAHL search 2**

Results:199*

*The librarian who performed this search noted: “Cinahl is a bit problematic over 50 results. So, here is a link that I hope will work for you:”

<http://ezproxy.library.usyd.edu.au/login?url=http://search.ebscohost.com/login.aspx?direct=true&db=ccm&bquery=clinical+quality+N4+(register*+or+registr*)&type=1&searchMode=Standard&site=ehost-live>

This link shows 227 entries as at 13 Mar 2021 as the screen shot below shows. The number at the time of Search 2 (30 March 2020) was 199.


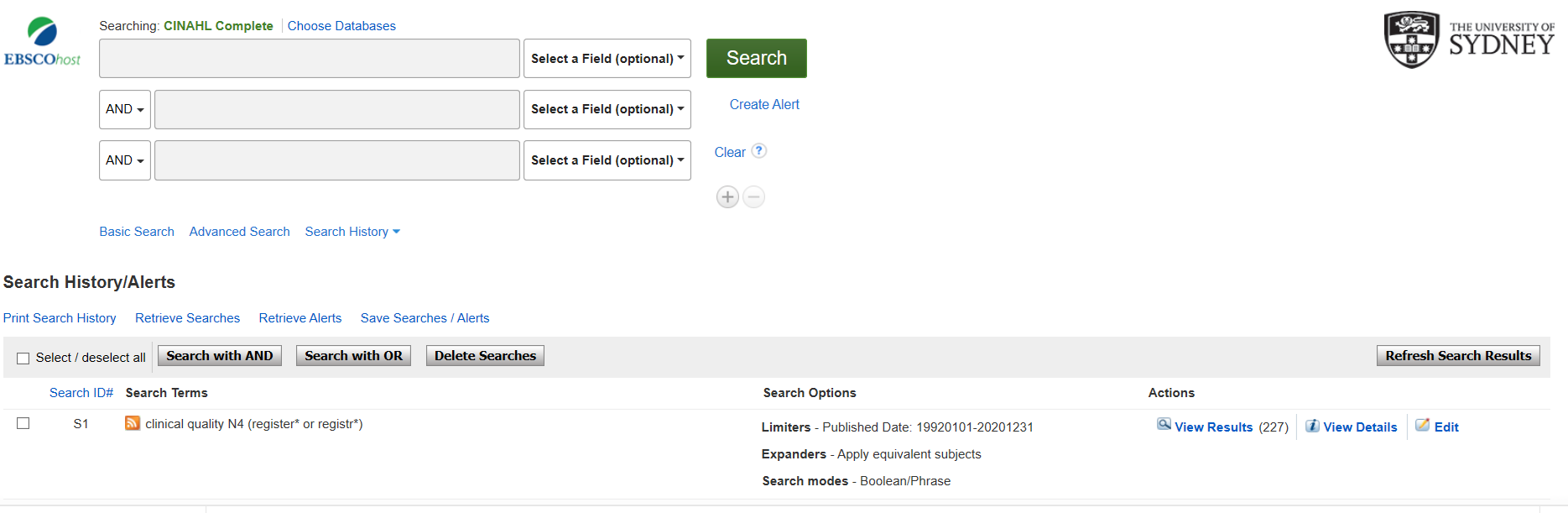

Supplement: Supplementary file 1 — Additional file 1. Literature search strategies [file 13643_2022_2104_MOESM1_ESM.docx]
