# Supplementary material for: Which strategies support the effective use of clinical practice guidelines and clinical quality registry data to inform health service delivery? A systematic review
Source: Syst Rev. 2022 Nov 9;11:237. doi: 10.1186/s13643-022-02104-1 (PMC9644489; doi:10.1186/s13643-022-02104-1)
Supplement: Supplementary file 2 — Additional file 2. Grey literature [file 13643_2022_2104_MOESM2_ESM.docx]

**Grey literature**

KD used sources listed in the original review template to guide the search of relevant grey literature and expanded this list using a ‘snowballing’ approach of checking relevant references and citations. The starting point for this search was the Australian Health Ministries Advisory Council (AHMAC) and Australian Commission on Safety and Quality in **Health Care** (ACSQHC) strategy document.^1^

**Findings**

The following list of grey literature was checked. It comprised mostly registry-based websites.

Agency for Clinical Innovation. About the Clinical Initiatives Leading Better Value Care program [includes reference to the Register of Outcomes, Value and Experience (ROVE)]. http://www/eih.health.nsw.gov.au/lbvc/about

# Agency for Clinical Innovation. Clinical Guidelines.

# https://www.aci.health.nsw.gov.au/resources/clinical-guidelines

Agency for Healthcare Research and Quality (AHRQ)

https://www.ahrq.gov/cpi/about/profile/index.html

American Heart Association’s Get With The Guidelines Program Expands Globally

https://newsroom.heart.org/news/american-heart-associations-get-with-the-guidelines-program-expands-globally [accessed 21 March 2020].

Australian Clinical Trials Alliance (ACTA). Registries Special Interest Group.
https://clinicaltrialsalliance.org.au/group/registries-special-interest-group/ [accessed 18 April 2020]

Australian Health Research Alliance. Health System Improvement and Sustainability initiative. Responses to questions circulated to Sydney Health Partners Research Leaders about models and methods to increase the use of clinical guidelines and registries [internal email].

Australian Institute of Health and Welfare. Australasian Association of Cancer Registries.
https://www.aihw.gov.au/about-our-data/our-data-collections/aacr

Australia and New Zealand Dialysis and Transplant Registry (ANZDATA)

<https://www.anzdata.org.au/>

Australian Breast Device Registry

https://www.abdr.org.au/

Australian Clinical Trials Alliance (ACTA). Registries Special Interest Group.

<https://clinicaltrialsalliance.org.au/group/registries-special-interest-group/>

Australian Clinical Outcomes Registry for Melanoma (MelCOR)
https://melanomaeducation.org.au/courses/melanoma48/ [accessed 6/5/2020]

Australian Commission on Safety and Quality in Health Care (ACSQHC). Framework for Australian clinical quality registries. Sydney: ACSQHC; 2014.

https://www.safetyandquality.gov.au/publications-and-resources/resource-library/framework-australian-clinical-quality-registries

Australian Commission on Safety and Quality in Health Care (ACSQHC). Prioritised list of clinical domains for clinical quality registry development: Final report. Sydney: ACSQHC; 2016

https://www.safetyandquality.gov.au/sites/default/files/migrated/Prioritised-list-of-clinical-domains-for-clinical-quality-registry-development-Final-report-Nov-2016.pdf

Australian Commission on Safety and Quality in Health Care (ACSQHC). Australian Register of Clinical Registries. Sydney: ACSQHC; 2020

https://www.safetyandquality.gov.au/our-work/national-arrangements-clinical-quality-registries#australian-register-of-clinical-registries

Australian Government. Department of Health. Draft National Clinical Quality Registry Strategy: Maximising the Potential of Australian Clinical Quality Registries (2019-2029)

https://www1.health.gov.au/internet/main/publishing.nsf/Content/Draft_National_%20CQR_Strategy

Australian Health Ministries Advisory Council (AHMAC) and Australian Commission on Safety and Quality in Health Care. Maximising the Potential of Australian Clinical Quality Registries. Using Data to Drive Improvements in Patient Care and Outcomes. A National Strategy 2019-2029 (Draft). Sydney: ACSQHC; May 2019.

file:///H:/SR%20for%20AHRA/Master%20list%20of%20grey%20literature_13April2020/ACSQHHC_Draft%20National%20CQR%20Strategy%20May%202019%20-%20Consultation.pdf

Australian Institute of Health and Welfare. Australasian Association of Cancer Registries

https://www.aihw.gov.au/about-our-data/our-data-collections/aacr

Australian Living Evidence Consortium. Frontier Projects
https://livingevidence.org.au/new-index-3

Australian Living Evidence Consortium. Medical Research Future Fund 2018-2020 Priorities Consultation. Submission to the Australian Medical Research Advisory Board. August 2018.

https://australia.cochrane.org/sites/australia.cochrane.org/files/public/uploads/LivingEvidence/aust_living_evience_consortium_mrff_priorities_2018-2020_final_for_web.pdf

Australian Register of Clinical Registries

https://www.safetyandquality.gov.au/australian-register-clinical-registries

Australian Stroke Clinical Register (AuSCR)
<https://auscr.com.au/>

Australian Stroke Data Tool (AuSDaT)
https://strokefoundation.org.au/Australian%20Stroke%20Coalition/AusDAT

Get With The Guidelines® Advances Health Services and Outcomes Research

Author: Paul Heidenreich, MD, MS. Pub Date: Friday, May 15, 2009
https://professional.heart.org/professional/ScienceNews/UCM_433092_Get-With-The-Guidelines-Advances-Health-Services-and-Outcomes-Research.jsp [Accessed 21 March 2020].

KHA-CARI Guidelines
Implementation overview. Ensuring the use of KHA-CARI Guidelines in clinical practice

http://www.cari.org.au/implementation/implementation_overview.html

Macquarie University Newsroom. Australian children receive healthcare in line with guidelines 60 per cent of the time: National study. 21 March 2018 [Newsroom report]

https://www.mq.edu.au/newsroom/2018/03/21/australian-children-receive-healthcare-in-line-with-guidelines-60-per-cent-of-the-time-national-study/

Medical Research Future Fund. 2018-2020 Priorities. Australian Living Evidence Consortium. Submission to the Australian Medical Research Advisory Board. August 2018
https://australia.cochrane.org/sites/australia.cochrane.org/files/public/uploads/LivingEvidence/aust_living_evience_consortium_mrff_priorities_2018-2020_final_for_web.pdf

Monash Clinical Registries
https://www.monash.edu/medicine/sphpm/registries

Monash Registry Special Interest Group
https://www.monash.edu/medicine/sphpm/registries/registrysig

Monash University Cancer Registries
https://www.monash.edu/medicine/sphpm/units/cancer-research

National Health and Medical Research Council. Recognised Health Research and Translation Centres.

https://www.nhmrc.gov.au/research-policy/research-translation/recognised-health-research-and-translation-centres

National Institute for Health and Care Excellence (NICE)

https://www.nice.org.uk/

Register of Outcomes, Value and Experience (ROVE)

https://www.health.nsw.gov.au/annualreport/Publications/2018/nsw-health-organisations.pdf

South Australia Clinical Cancer Registry (SACCR)

https://www.sahmriresearch.org/our-research/sahmri-registry-centre/south-australia-clinical-cancer-registry-saccr

South Australian Health and Medical Research Institute (SAHMRI)

https://www.sahmriresearch.org/our-research/sahmri-registry-centre

Sutherland, Kim. Focusing on unwarranted clinical variation to assess performance. Bureau of Health Information [blog].

www.bhi.nsw.gov.au/About_BHI/Blog/authors/kim/Focusing-on-unwarranted-clinical-variation-to-assess-performance
