# Supplementary material for: Which strategies support the effective use of clinical practice guidelines and clinical quality registry data to inform health service delivery? A systematic review
Source: Syst Rev. 2022 Nov 9;11:237. doi: 10.1186/s13643-022-02104-1 (PMC9644489; doi:10.1186/s13643-022-02104-1)
Supplement: Supplementary file 3 — Additional file 3. Critical appraisal using MMAT [file 13643_2022_2104_MOESM3_ESM.docx]

**Critical appraisal of included articles as assessed by MMAT^1^**

| **Study design** | **Author, year** | **CPG** | **CQR** | **Question 1** | **Question 2** | **Question 3** | **Question 4** | **Question 5** |
| --- | --- | --- | --- | --- | --- | --- | --- | --- |
| **Qualitative studies** |  | | | Is qualitative approach appropriate to answer research question? | Are qualitative data collection methods adequate to address research question? | Are the findings adequately derived from the data? | Is interpretation of results sufficiently substantiated by data? | Is there coherence between qualitative data sources, collection, analysis and interpretation? |
|  | Alguren 2019 |  | x | Yes | Yes | Yes | Yes | Yes |
|  | Egholm 2019b |  | x | Yes | Yes | Yes | Yes | Yes |
|  | Granstrom 2017 |  | x | Yes | Yes | Yes | Yes | Yes |
|  | Norman 2020 |  | x | Yes | Yes | Yes | Yes | Yes |
| **Quantitative RCT** |  | | | Is randomisation appropriately performed? | Are the groups comparable at baseline? | Are there complete outcome data? | Are outcome assessors blinded to the intervention provided? | Did the participants adhere to the assigned intervention? |
|  | Hendriks 2012 | x |  | Yes | Yes | Yes | Yes | Yes |
| **Quantitative**  **non-randomised** |  | | | Are the participants representative of the target population? | Are measurements appropriate for outcome and intervention (or exposure)? | Are there complete outcome data? | Are the confounders accounted for in the design and analysis? | Is the intervention administered (or exposure occurred) as intended? |
|  | Cadilhac 2017 |  | x | No | Yes | Yes | Yes | Yes |
|  | Larson 2018 | x |  | Yes | Can’t tell | No | Can’t tell | Yes |
|  | Moen 2019 | x |  | Yes | Yes | Yes | Yes | Yes |
|  | Mor 2000 | x |  | Yes | Yes | Can’t tell | Can’t tell | Can’t tell |
|  | Nag 2019 |  | x | No | Can’t tell | Can’t tell | Can’t tell | Can’t tell |
|  | Rutledge 2018 | x |  | Yes | Yes | Yes | Yes | Yes |
|  | Stark 2013 | x |  | Yes | Yes | Yes | Yes | Yes |
| **Quantitative descriptive** |  | | | Is sampling strategy relevant to address the research question? | Is sample representative of the target population? | Are the measurements appropriate? | Is the risk of nonresponse bias low? | Is the statistical analysis appropriate to answer the research question? |
|  | Alguren 2018 |  | x | Yes | Unclear | Yes | No | Yes |
|  | Egholm 2019a |  | x | Yes | No | Yes | Yes | Yes |
|  | Eldh 2016 |  | x | Yes | Yes | Yes | Yes | Yes |
|  | Lipitz-Snyderman |  | x | Yes | Can’t tell | Can’t tell | No | Yes |
|  | Viktrup 2004 | x |  | Yes | Can’t tell | Can’t tell | No | Yes |

1. Mixed Methods Appraisal Tool (MMAT), Version 2018
