# Supplementary material for: Which strategies support the effective use of clinical practice guidelines and clinical quality registry data to inform health service delivery? A systematic review
Source: Syst Rev. 2022 Nov 9;11:237. doi: 10.1186/s13643-022-02104-1 (PMC9644489; doi:10.1186/s13643-022-02104-1)
Supplement: Supplementary file 4 — Additional file 4. Critical appraisal using QI-MCQS [file 13643_2022_2104_MOESM4_ESM.docx]

**Critical appraisal of included articles as assessed by QI-MCQS^1^**

| **Questions** | **Ahern 2020** | **Cadilhac 2019** | **Dyrkorn 2012** | **O’Grady 2007** |
| --- | --- | --- | --- | --- |
| 1. Organisational motivation: Consider quality of care problems, organisational problems, regulations, legal constraints and external financial incentives at the target organisation. | Met | Met | Met | Met |
| 2. Intervention rationale: Consider citations of theories, logic models, or existing empirical evidence that links the intervention to its expected effects. | Met | Met | Met | Met |
| 3. Intervention description: Consider details describing change in delivery of care, provider behaviour or organisational structure needed to replicate the intervention including the involved key personnel. | Met | Met | Met | Met |
| 4. Organisational characteristics: Consider environment (urban/rural, academic/non-academic), type of care (e.g., primary care), size of the organisation, patient mix, staff mix or reimbursement type. | Met | Met | Met | Not met |
| 5. Implementation: Consider types of staff involved, activities or methods used such as pilot testing or Plan-Do-Study-Act cycles, staff education, and involvement of stakeholders in introducing the intervention. | Met | Met | Met | Met |
| 6. Study design: Consider type of evaluation and how the authors evaluated whether the intervention worked. | Met | Met | Met | Met |
| 7. Comparator: Consider details about the control group or pre-intervention status quo e.g., existing standard of care, routine care or care processes used in the control group. | Met | Met | Met | Met |
| 8. Data source: Consider data sources (e.g., routine hospital data, data collected by the study investigator), data collection method and how outcome of interest is defined. | Met | Met | Met | Met |
| 9. Timing: Consider clarity of the timeline of the intervention and a clear indication of whether baseline data was present. | Not met | Met | Met | Met |
| 10. Adherence: Consider reporting of compliance with intervention for the duration of the study, fidelity data on intervention use, or described mechanisms that ensures compliance. | Not met | Met | Not met | Met |
| 11. Health outcomes: Consider patient and non-professional care-giver health-related outcomes (including quality of life), but exclude satisfaction, provider-behaviour (e.g., number of diagnostic tests ordered, knowledge) and process improvements. | Not met | Not met | Met | Met |
| 12. Organisational readiness: Consider reported QI resources and culture (e.g., existing QI committee, leadership commitment, prior QI experience, staff attitudes, and education and decision support resources) and results of barriers and facilitator assessments. | Met | Not met | Met | Met |
| 13. Reach: Consider number of units or sites participating in intervention compared to the available / eligible units (e.g., the number of participating sites without knowing how many sites were initially approached / were eligible is not sufficient). | Met | Met | Met | Met |
| 14. Sustainability: Consider discussions of sustainability, reference to organisational resources (e.g., costs and necessary commitments) and policy changes needed to sustain the intervention after withdrawal of study personnel and research resources, evidence of enduring changes (e.g. automated electronic reminders), or an extended duration of the intervention period as evidence of sustainability. | Met | Met | Met | Met |
| 15. Spread: Consider evidence of spread or failure to spread and large rollouts; available resources such as toolkits, how-to manuals, protocols, or booklets that describe the intervention in detail and could facilitate spread and replication; or discussions of spread potential. | Met | Met | Met | Met |
| 16. Limitations: Consider whether the interpretation of the reported findings takes the study design (e.g., the lack of comparator) or other evaluation limitations into account; refers to the presented data (not future research / developments or intervention limitations) | Met | Met | Not met | Met |

1. Quality Improvement Minimum Quality Criteria Set (QI-MQCS) – Version 1.0
