# Supplementary material for: Which strategies support the effective use of clinical practice guidelines and clinical quality registry data to inform health service delivery? A systematic review
Source: Syst Rev. 2022 Nov 9;11:237. doi: 10.1186/s13643-022-02104-1 (PMC9644489; doi:10.1186/s13643-022-02104-1)
Supplement: Supplementary file 5 — Additional file 5. CPG results [Clinical Practice Guideline results] [file 13643_2022_2104_MOESM5_ESM.docx]

**Additional File 5. Clinical practice guidelines: Findings from included studies**

| **First author, location, year** | **Study Objectives, Benchmarking?** | **Type of guideline** | **Type of registry** | **Healthcare findings** | **What works?** |
| --- | --- | --- | --- | --- | --- |
| Dyrkorn  Norway 2012 | - To reduce rate of caesarean wound infections to <8%  - Benchmarking of local caesarean infection rates against national data. | - Cochrane review of techniques for caesarean section was used to ensure that the procedures were in accordance with the best available evidence for reducing risk of wound infections. | - Norwegian Surveillance  System for Hospital-acquired Infections (NOIS). (participation is mandatory for all Norwegian hospitals). | - 47 cases of surgical wound infection were identified, all diagnosed after discharge from hospital and all characterised as superficial skin infections.  - After year 1 (2009), infection rates down to 2.6% but back up to 6.4% in year 2. Reinforcement of intervention measures resulted in a further reduction in the infection rate 1.1% in 2010.  - Found a significant (p<0.004) increase in median operation time following the intervention from 31-37 minutes.  - No significant differences between the groups in elective surgery rate, patient age or follow-up rate. | - Frequent reporting of results to the Head of the Maternity Clinic, frequent meetings with the improvement team, and targeted communication with relevant staff.  - Surveillance should be continuous.  - Leadership involvement essential for success.”  - No adherence data for different components of the intervention which were introduced simultaneously. |
| Hendriks Netherlands 2012 | - To compare t­he AF clinic with routine care in patients with AF.  - 356 patients in each group.  - Follow-up 12 months+ - No benchmarking. | - Referenced 2006 Guidelines for the management of patients with AF. (American College of Cardiology/  American Heart Association Task Force on Practice Guidelines and the European Society  of Cardiology Committee for Practice Guidelines). | N/A | Nurse-led care superior to usual care in terms of:  - primary outcome (composite of cardiovascular hospitalisation and mortality): 14.3% vs 20.8%; [HR: 0.65; 95% CI: 0.45–0.93; P <0.017].  - Rate of cardiovascular death: 1.1% vs 3.9%; [HR: 0.28; 95% CI: 0.09–0.85; P < 0.025].  - Number of cardiovascular hospitalisations: 13.5% vs. 19.1% [HR: 0.66; 95% CI: 0.46–0.96). Adjusted HR: 0.64; 95% CI 0.44–0.93.  - Adherence to guideline recommendations  - Relative risk reduction of 35%  - Patients better informed about their disease and its management. | - Comprehensive intervention focusing on patient education, reassurance, prophylactic measures guided by electronic decision support based on the guidelines, time spent with the patients, and teamwork between the nurse specialist and the cardiologist.  - Use of an electronic patient record with incorporated dedicated  decision support software based on the guidelines may downsize complexity and improve adherence to recommendations.  - Multi-disciplinary approach with cardiologists and nurses working closely together and having to justify reciprocally deviations from the protocol may largely preclude treatment decisions which do not comply with the guidelines. |

| **First author, location, year** | **Study Objectives, Benchmarking?** | **Type of guideline** | **Type of registry** | **Healthcare findings** | **What works?** |
| --- | --- | --- | --- | --- | --- |
| Kamišali´c  Spain/Slovenia 2018 | - Analyse and classify the sorts of time constraints in medical processes.  - Propose and use formalisms to enable automatic generation of temporal models from clinical data.  - Study adherence of these intervention models to CPG recommendations. | Two CPGs for Arterial Hypertension, Heart Failure and Stable Ischaemic Heart Disease. | N/A | - No mechanisms to help physicians obtain evidence-based knowledge about the time constraints that should be included in CPGs.  - Medical records and health care information systems are promising sources of information to detect medical knowledge about time in medicine. | - Knowledge from clinical experiences can be captured with two types of structures (micro- and macro-temporality) that have been shown to describe temporal constraints in the management of cardiovascular diseases.  - These structures can be easily understood by clinicians.  - Application of three proposed algorithms confirms clinical data can be transformed into temporal knowledge to manage new chronic patients, to audit clinical actions performed in health care centres, and to analyse adherence of clinical actions to standard procedures described in CPGs.  -  Timed Medical Decision Support Systems (TMDSS) development is feasible. |
| Larson  USA 2018 | To determine if: - academic detailing made prescribing behaviours more consistent with guidelines;  - which participant and intervention characteristics were associated with adoption of Prescription Monitoring Program (PMP) use. - No benchmarking. | Not stated but referenced the following: Clinical guidelines from American Pain Society & American Academy of Pain Medicine on use of opioid therapy in chronic noncancer pain. | State-based PMP containing data on patient prescription history. Prescriber registration with PMP is voluntary in some US states and mandatory in others. | - 83% of physicians who were nonusers at pre-intervention were PMP adopters at follow-up; (McNemar, P < .001)  - Mean frequency rating significantly increased from preintervention to follow-up (3.2 vs. 3.8, P < .001); (McNemar, P < .001).  - A single visit with a trained academic detailer was effective in changing some, but not all, physician opioid prescribing behaviours and substantially increased utilisation of PMP. | - Single academic detailing visit appears to increase guideline-consistent behaviour among a group of physicians not mandated to register or use the PMP.  - No long-term data on adherence to guidelines. |
| Moen  USA  2019 | - To examine how the connectedness of physicians and hospitals, measured using network analysis, relates to guideline-consistent ICD implantation. | Not stated | National Cardiovascular Data Registry (NCDR) ICD Registry. (A Center for Medicare and Medicaid Services (CMS)-mandated hospital registry for the in-patient setting). | - Patients **less** likely to meet guidelines if their referring hospital had more connections to other hospitals (OR = 0.49, 95% CI, 0.25 – 0.96).  - Patients **more** likely to meet guidelines if their ICD surgery hospital had more connections (OR=1.61, 95% CI, 0.98 – 2.64). | - ICD surgeon’s network measures were **not** associated with guideline-consistent implementation.  - These associations, taken together, “are consistent with the idea that regionalisation of specialised services, in which peripheral hospitals refer to regional centers (“hub and spoke”) are associated with greater guideline adherence.” |

| **First author, study location, year** | **Study Objectives, Benchmarking?** | **Type of guideline** | **Type of registry** | **Healthcare findings** | **What works?** |
| --- | --- | --- | --- | --- | --- |
| Mor USA  2000 | - To evaluate the impact of surgeon-specific “performance reports” on adherence to treatment guidelines for older women with breast cancer.  - To evaluate the feasibility and effectiveness of instituting locally derived breast cancer treatment guidelines in community hospitals. | Locally derived breast cancer treatment guidelines. | Hospital tumour registries | - Patients over 80 with breast-conserving surgery were significantly less likely to undergo radiation therapy (AOR = 0.08 [0.04, 0.14]) or appropriate adjuvant therapies (AOR = 0.14 [0.08, 0.22]) or both, relative to 70- to 79-year-old patients.  - This effect did not improve post-intervention.  - Mastectomy patients under 70 years old were significantly more likely (AOR =2.37), and women 80 or over were somewhat less likely (AOR = 0.84), to receive appropriate hormonal or chemotherapy treatment, or both, relative to those aged 70 to 79 years. | - Much variability in compliance with guidelines, but surgeons’ characteristics **did not** explain this variation.  - Providing surgeons with “feedback” on the appropriateness of adjuvant treatment for older patients was **insufficient** to alter established practices. - Using guideline compliance data as standard “quality indicators” of physician practice may be required. |
| O’Grady  USA  2007 | - Regular audits of private medical oncology practices conducted by Fox Chase Cancer Centre Program (FCCCP)  - Benchmarks quality indicators and guidelines. | National Comprehensive Cancer Network (NCCN) Clinical Practice Guidelines in Oncology.  Developed through a collaboration of 21 National Cancer Institute (NCI)-designated cancer centres and updated annually, these guidelines contain extensive algorithms for treatment decision-making and encompass 95% of cancer diagnoses. | Tumour registry for disease-specific data | - FCCCP has conducted successful audits of gastric, colorectal, and breast cancer.  - The 2005 stage II/III breast cancer evaluation had a mean compliance per parameter of 88%, with 15 of 16 practices achieving mean compliance greater than 80%.  - Recent evaluation of localised breast cancer shows high compliance with guidelines and identifies areas for focused education. | - FCCCP has developed rigorously defined metrics that can be realistically implemented and monitored.  - A large-scale quality assurance audit in a community cancer partner network is feasible.  - Partnership between academic and community oncologists produces a quality review process that is broadly applicable and adaptable to changing medical knowledge. |

| **First author, location, year** | **Study Objectives, Benchmarking?** | **Type of guideline** | **Type of registry** | **Healthcare findings** | **What works?** |
| --- | --- | --- | --- | --- | --- |
| Paxton  USA  2012 | - To describe the Kaiser Permanente (KP) implant registries  - Benchmarking through reports of medical centre- and surgeon-specific profiles. | - Registries verify adherence to national practice guidelines, such as indications for ICD implantation as established by the Ameri­can Academy of Cardiology.  - Also use formulary device guidelines. | Eight orthopaedic and cardiac implant registries. | - Enhanced patient safety through identification of affected pa­tients during major recalls, identification of risk factors, development of risk calculators, and surveillance programs for infec­tions and adverse events.  - Effective QI activities include changes in practice related to registry information output.  - Cost-effectiveness strategies include collaborations with sourcing and contracting groups, and assistance in adherence to formulary device guidelines.  - Research studies using registry data included postoperative complications, resource utilisation, infection risk factors, thromboembolic prophylaxis, effects of surgical delay on concurrent injuries, and sports injury patterns. | - Integrated health care system, ad­ministrative databases, and comprehen­sive health electronic records.  - Use of risk calculators that allow patients and surgeons to make clinical decisions at the point of care. Surgeons have access to these prognostic tools via the internal KP Web site.  -Quarterly quality reports for monitor­ing infection and other complications at the medical centre level.  - Wide dissemination of registry findings about clinical best practices or quality improvement opportunities.  - Medical centre-specific results and surgeon profiles. These targeted reports can be used to compare information among locations, Regions, and even nationally, creating an opportunity for benchmark­ing and learning.  - De­vice performance evaluations comparing similar implants and patient and surgical characteristics. These analyses are critical in new technology adoption or for cost-benefit analysis of similar implants, or both. |
| Rutledge  Australia  2018 | - To reduce widespread overuse of CT and whole-body bone scans staging investigations for men with newly diagnosed prostate cancer in the Hunter region of  NSW. | - Primary guidelines were the American Urological Association and European Association of Urology guidelines. | N/A | - In low-risk patients, the use of CT decreased from 43% to 0% (P = 0.01).  - 21% of patients had bone scans in the pre-intervention group compared with 18% in the post-intervention  group (P = 0.84).  - In intermediate-risk patients, the use of CT decreased from 89% to 34% (P < 0.001) and the use of bone scan decreased from 63% to 37% (P = 0.02).  - In high-risk patients, the appropriate use of imaging was  maintained, with CT performed in 87% compared with 85% and bone scan in 87% compared with 65% (P = 0.07).  - Imaging use in accordance with guidelines yielded few positive results for metastatic disease in low- and intermediate-risk patients. | - A focused, clinician-centred education program can lead to improved guideline adherence at a regional level.  - Adherence to imaging recommendations increased  significantly in low- and intermediate-risk patients in the post-intervention group and an appropriately high proportion of patients with high-risk continued to have CT imaging. |

| **First author, study location, year** | **Study Objectives, Benchmarking?** | **Type of guideline** | **Type of registry** | **Healthcare findings** | **What works?** |
| --- | --- | --- | --- | --- | --- |
| Stark Germany  2013 | - To examine whether CHD-DMPs increase the frequency of guideline-care  - and whether CHD-DMPs and guideline-care improve survival over 4 years.  - No benchmarking. | Guideline-care was  based on patient reports regarding medical advice (smoking, diet, or exercise) and prescribed medications. | KORA (Cooperative Health Research in the Augsburg Region) Myocardial Infarction Registry. | - CHD-DMP participation increased the likelihood of receiving guideline-care (OR 1.55, 95 % CI 1.20; 2.02)  - CHD-DMP participation did not significantly improve survival (HR 0.90, 95 % CI 0.64–1.27).  - Guideline-care significantly improved survival (HR 0.41, 95 % CI 0.28; 0.59). | - CHD-DMP participants more frequently reported appropriate medical care and were more likely to receive guideline-care.  - Individual guideline-care components that  significantly improved survival were beta-blockers, statins, and platelet aggregation inhibitors.  - CHD-DMPs increase the likelihood of guideline care and that guideline care is the important component of CHD-DMPs for increasing survival.  - Need for RCT design to clarify the role of specific components. |
| Viktrup  Denmark  2004 | - To identify the management of UI in general practice after distribution of clinical guidelines and  reimbursement for using a UI diary. | - July 1999: UI in general practice.  - September 2001: UI in female, geriatric, or neurological patients. | National Health Service County Registry. | - 87% had read the guidelines but only 47% used them daily.  - 69% had read and appreciated other UI guidelines distributed prior to the study.  - 83% sometimes or often actively asked their patients about UI.  - 92% sometimes or often included a voiding diary in the UI assessment.  - 35% (85/243) received reimbursement within 12 months after the introduction of payment for using a voiding diary. | - Available registry data concerning voiding diary reimbursement, prescribed UI drugs, UI consultations in outpatient clinics, and patient reimbursement for pads were **insufficient or too variable** to determine significant trends.  - Concluded that “Using a questionnaire or data from health registries does not seem to provide reliable information.” |
