# Supplementary material for: Which strategies support the effective use of clinical practice guidelines and clinical quality registry data to inform health service delivery? A systematic review
Source: Syst Rev. 2022 Nov 9;11:237. doi: 10.1186/s13643-022-02104-1 (PMC9644489; doi:10.1186/s13643-022-02104-1)
Supplement: Supplementary file 6 — Additional file 6. CQR results [Clinical Quality Registry results] [file 13643_2022_2104_MOESM6_ESM.docx]

| **First author, location, year** | **Study Objectives, Benchmarking?** | **Type of registry** | **Healthcare findings** | **What works?** |
| --- | --- | --- | --- | --- |
| Ahern  Australia  2020 | - To identify clinician and unit participation in clinical registries.  - To investigate opportunities to engage clinicians to share clinical registry information within the organisation,  - To integrate CQR reporting within the health service clinical governance framework. | 69 clinical registries or external audits that Alfred Hospital participated in. | Outcomes comprised:  - the establishment of a CQR interest group  - development of a CQR reporting guideline  - a calendar of CQR site reports;  - creation of a CQR dashboard to monitor site CQR outcomes over time.  - Investment in both local and system-wide initiatives required to ensure valuable clinical information (datasets including EMRs, administrative data and government/ agency reports) is recognised and maximally used. | - A project officer to engage with clinicians in one-on-one meetings allowed a deeper understanding of the history, purpose and significance of each registry. - Acknowledging the barriers, time and effort required for clinicians to input data into registries was a key focus of individual meetings, as was creating a culture of engagement.  - Local CQR clinician champions were critical in highlighting the benefits of sharing CQR results within the organisation.  - Champions led by example, incorporating registry reports and feedback into regular local and organisation-wide audits and presentations. |
| Algurén  Sweden  2018 | - To examine the regular use of quality indicators from two Swedish cardiovascular National Quality Registries (NQRs) by nurses and physicians. | Two Swedish cardiovascular NQRs: (a) Swedish Heart Failure Registry (SwedeHF) and (b) Swedeheart. | - Survey revealed that NQRs were rarely integrated regularly in clinical work for QI.  - Most respondents used quality indicators infrequently (<3 times/year).  - Quality indicators used mainly for producing healthcare activity statistics.  - Physicians used the NQRs significantly more than nurses (18 vs. 7.5 times/year; P < 0.000)  - Nurses entered data, while physicians used data for QI (training materials the same).  - Median use of Swedeheart was six times greater than SwedeHF (P < 0.000).  - Around twice as many Swedeheart users had data analysis and QI roles compared to SwedeHF users. | - Linking registration of quality indicators to using them for QI activities increases their routine use and makes them meaningful tools for professionals.  - Essential to adapt quality measurement efforts to the needs of end-users so they can use indicators to develop and evaluate QI activities and contribute to better patient outcomes.  - Clinicians motivated to use the registries found this work meaningful, and believed it contributed to their own learning to a large extent. |

**Additional File 6. Clinical quality registries: Findings from included studies**

| **First author, location, year** | **Study Objectives, Benchmarking?** | **Type of registry** | **Healthcare findings** | | **What works?** |
| --- | --- | --- | --- | --- | --- |
| Algurén  Sweden  2019 | To compare 12 teams from two QICs using NQRs from different areas of healthcare—heart failure and osteoarthritis— and compare characteristics and activities in detail in relation to their changed outcomes from baseline to 3 years. | Swedish Heart Failure Registry (SwedeHF) and the Better Management of Patients with OsteoArthritis Registry (BOA) | - Heterogeneous outcomes within teams, across teams and across the two QICs.  - Common activities included: increased availability of, and more structured, follow-up; improved guidelines and care processes; better information for, and improved dialogue between, patients and colleagues.  - SwedeHF-QIC program cost was five times higher than BOA, included more experts, project time was twice as long and there were four times as many learning seminars.  - No clear differences in number of improvements of chosen goal indicators between SwedeHF and BOA.  - Participants appreciated the programs and perceived they had contributed to their own learning. | | - Results indicate no linear relationship between QIC designs and sustained improvements on outcomes.  - Activities focusing on adherence to standard care programmes and on increased follow-up of patients seemed to lead to more long-lasting outcome improvements. |
| Cadilhac  Australia  2017 | - To improve discharge care post-hospital admission for stroke  - Change in performance gap score  - 12 months follow-up | Australian Stroke Clinical Registry (AuSCR) | - Significant improvements in adherence to discharge processes  - A non-significant decay effect at 12 months (composite outcome: 89% post-intervention vs 85% sustainability period, p=0.08).  - Improved adherence at both sites between baseline (hospital selection) and pre-intervention phase (net change in composite outcome site 1: 42% and site 2: 16%).  - Improvements from pre-intervention adherence were: antiplatelet therapy  (88%vs96%, p=0.02); antihypertensive prescription (61%vs79%, p<0.001); discharge planning (72%vs94%, p<0.001); composite outcome (73%vs89%, p<0.001).  - Marked variability between sites: (net change in composite outcome site 1: 7% and site 2: 33%) | | - Improving discharge care in hospitals through a staged, evidence-based, multifaceted intervention was effective and sustainable.  - Recognition that existing processes and administrative systems could be leveraged by changing workflow processes.  - Providing example of a comprehensive discharge care plan for clinicians to refer to.  - Using reminders (e.g. stickers in medical records) to facilitate medication prescriptions.  - Regular reviews of data at team meetings. |
| Cadilhac  Australia  2019 | - To assess impact of multicomponent program (financial incentives and quality improvement interventions) on stroke care processes. | Australian Stroke Clinical Registry (AuSCR). | - 18% improvement in median composite score for adherence to ≤8 indicators (95% CI, 12%–24%).  - Largest improvement from financial incentives (14%; 95% CI, 8%–20%),  - Indicators addressed in action plans provided an 8% improvement (95% CI, 1%–17%).  - National score (4 indicators) improved by 17% (95% CI, 13%–20%) versus 0% change in other Australian hospitals (95% CI, −0.03 to 0.03).  - Access to stroke units improved more in Queensland than in other Australian hospitals (P<0.001). | | -QI interventions significantly improved clinical practice.  - Findings primarily driven by financial incentives, but externally facilitated, quality improvement workshops also contributed.  - Data suggest that multicomponent and complementary interventions are more effective than single-component ones.  - Initial targeting with financial incentives associated with concurrent improvements in adherence to some measured clinical indicators (i.e. thrombolysis for acute ischemic stroke and early mobilisation), but not all. |
| **First author, location, year** | **Study Objectives, Benchmarking?** | **Type of registry** | **Healthcare findings** | **What works?** | |
| Eccleston  Australia  2017 | - To determine if measurement and local reporting of data improves patient outcomes through improving compliance with guideline therapies.  - Key performance outcomes benchmarked against the aggregated study cohort and international standards. | Genesis Cardiovascular Outcomes Registry percutaneous coronary intervention (GCOR-PCI). | - Early data identified specific practice patterns associated with lower rates of statin therapy post-PCI.  - Significant improvement in the rates of statin therapy at discharge (92.1 vs. 94.4% p<0.03) and 12 months post-PCI (87.0 vs. 92.2% p<0.001) and of antiplatelet therapy at 12 months (90.7 vs. 94.3% p<0.001).  - Found very high procedural success rates, low in-hospital complication rates, incomplete provision of evidence-based therapies with wide regional variation and low long-term cardiovascular event rates following PCI for either acute coronary syndrome (ACS) or stable coronary artery disease (CAD) in Australian Private hospitals. | - “Introducing a Registry to document medication use and outcomes after PCI was associated with improvements in compliance with statin and anti-platelet therapy after PCI.”  - Infers improvement in patient outcomes due to the establishment of a registry. | |
| Egholm  Denmark  2019a | - To investigate use of data from cardiac rehabilitation CQR, specifically:  - Extent and facilitators of data used for local QI  - Differences in use between frontline staff and managers.  - Data reported on a local, regional and national level for benchmarking. | Danish Cardiac Rehabilitation Database | - Reports of registry use at department level  varied significantly between groups of respondents (p=0.006).  - Managers’ use of registry data was associated with data quality and usefulness (regression coefficient=0.43, p=0.027)  - Frontline staffs’ use was associated  with management involvement in QI work (regression coefficient=0.90, p=0.017) and personal motivation (regression coefficient=1.66, p<0.001). | - Findings suggest relatively sparse use of data in local QI work.  - Resources, such as time and competencies, and receiving support were **not** associated with use of registry data (see comments).  - Nearly half of non-responders (mostly managers, particularly heads of departments) stated that they could not respond due to lack of knowledge of the registry. | |
| Egholm  Denmark  2019b | - To explore how staff perceive the implementation process cardiac rehabilitation registries in England and Denmark. | The national cardiac rehabilitation CQRs in the UK (mature, voluntary registry) and Denmark (relatively new, mandatory). | - Registry implementation is complex.  - Implementation received little focused attention.  - Although data linkage was supposed to be a facilitator for registry use in the Danish registry, poor execution led to frustrations and demotivation.  - Very little formal planning of implementation was reported in either country  - Lack of management involvement or support  - Not all informants were aware of the purpose of the registries, and/or were lacking  resources and know-how to use data.  - Very few respondents reported actual use of data to improve care.  - Ambiguity of variables was a source of frustration.  - Need to ensure adequate resources and staff competencies.  - Registry organisations need secure, ongoing funding. | - Important to assess quality of source registry and perform thorough testing before data linkage is implemented.  - Tying data entry to existing routines makes it easier (same day entry).  - Room for improvement of the registries’ user-friendliness to better fit multiple different practice processes, and thus facilitate registry use.  - Champions played a vital role.  - Management support in the data collection and entry phase is crucial.  - Developing a culture of data  reporting may be important in an CQR implementation perspective. | |

| **First author, location, year** | **Study Objectives, Benchmarking?** | **Type of registry** | **Healthcare findings** | **What works?** |
| --- | --- | --- | --- | --- |
| Eldh  Sweden  2016 | - To investigate facilitators and barriers to use of registry data in clinical quality improvement. | Swedish Stroke Registry (Riksstroke) | - 88% of respondents thought the use of Riksstroke data facilitated identification of stroke care improvement needs.  - 78% acknowledged that their data motivated quality improvements (78%).  - Use of Riksstroke data for QI initiatives was associated (R^2^ =0.76) with ‘Colleagues’ call for local results’ (p=<0.001), management requests for Registry data (p=<0.001), and being simple to explain to colleagues (p=0.02). | - Most participants considered Riksstroke to enable comparisons using relevant and reliable data, and resources spent on Riksstroke to be worthwhile.  - Data analyses and QIs based on the data received less attention than data registration.  - Use of Riksstroke data for QI initiatives was strongly related to the interest and engagement of fellow stroke care staff and managers.  - Implementing automatised data capture could shift resources from securing data to data-led quality improvement work. however,  - Health professionals, managers and policymakers need further support and opportunities to engage in joint ventures. |
| Granström  Sweden  2018 | - To investigate support strategies of regional Quality Research Centres (all NQRs are connected to 1 of 6 QRCs). | National Quality Registries (NQRs) | - Quality Research Centres (QRCs) used a range of strategies to involve NQRs in local improvement projects.  - These included improvement teams, learning seminars, small‐scale testing, and the use of PDSA cycles.  - Several QRCs also used coaching methods and some used pre-existing change concepts or collaborated with local development units.  - A gap exists between national agencies and the decentralised autonomous healthcare system in Sweden.  - A main challenge was that QRCs have a national mandate to create synergies between registries and serve as a link between national levels and NQRs, but are not part of the formal healthcare organisational structure. | - QRCs were able to apply for targeted project funding from the national initiative to develop and test strategies for increased use of NQRs in clinical improvement work.  - No evaluation of what works and what doesn’t – authors argue it is context dependent.  -  Support functions with mixed and complex missions have to use a variation of strategies to reach relevant actors and achieve changes.  - A top‐down focus on regulatory conformity needs to be complemented by a bottom‐up investment in the local improvement capacity in healthcare organisations. |

| **First author, location, year** | **Study Objectives, Benchmarking?** | **Type of registry** | **Healthcare findings** | **What works?** |
| --- | --- | --- | --- | --- |
| Klaiman  USA  2014 | - To study effective tools and strategies in the design and use of 12 clinical registries.  - Examples of benchmarking include Society of Thoracic Surgeons (STS) Adult Cardiac Surgery Registry that provides information for public reports that rank cardiac surgery groups. | 3 US cancer registries, 3 US cardiovascular registries, 2 Canadian maternity registries and 4 joint replacement registries (2 US, 1 Swedish, 1 Canadian). | - 12 effective registries were successful in one or more of 6 key areas: data standardisation, transparency, accuracy/ completeness of data, participation by providers, financial sustainability, and/or providing feedback to providers.  - Rare for registries to have all 6 elements necessary for effectiveness.  - Focusing on 1 or 2 key elements can help registries be better leveraged for improving health care performance.  - Cardiology registries best at QI and transparency through use of data integrators, establishing partnerships between professional societies in registry design and implementation, implementing creative mechanisms for registry financing, providing rapid feedback to providers, and using consumer-oriented products to share outcomes data. | - US **legal mandate** to report cancer cases encourages a high rate of participation among providers of cancer treatment.  **- Financial incentives**. Payers increasingly require cardiovascular registry participation as a condition of eligibility for bonus programs, preferred provider network status, and reimbursement.  **- Funding models.** CV registries have several ways to maintain financial stability, including subsidies from pharmaceutical and device manufacturers, dues paid by participating providers, and revenues from the sale of registry data and from grants/contracts for studies derived from those data.  - **Data linkage**. Directly report back on measures to Centres for Medicare and Medicaid Services and also to providers at the individual physician or group level and benchmark performance measures to national averages. |
| Lipitz-Snyderman  USA  2019 | - To report on frontline physician experiences within the Memorial Sloan Kettering Cancer Centre (MSK)  Cancer Alliance.  - MSK developed their own standards of care for benchmarking. | 3 individual cancer institutes’ tumour registries. | - Most respondents reported high value from Alliance activities that were applicable to them, such as attending MSK tumour boards (94%) and lecture series (96%).  - Across all respondents, most reported satisfaction with engagement opportunities, such as MSK physician participation in their institution’s meetings (76%).  - Most demand for more lecture series (45%). - Most respondents (88%) reported the Alliance had led to practice change, either for  themselves or for clinicians at their institution. - Many attributed this practice change to MSK disease-specific process measures. | - Activities most valued by community physicians were heavily physician relationship–based.  - A distinguishing feature of the MSK  Cancer Alliance is the development of standards of care by MSK multidisciplinary disease management teams, covering the treatment pathway from diagnosis to survivorship. |
| Løwer  Norway  2013 | - To describe the new Norwegian Surveillance System for Healthcare-Associated Infections’ (NOIS) module for surgical site infections (SSI).  - To evaluate the completeness of hospital participation, the effectiveness of automated data collection, and the added value of follow-up after hospital discharge. | The national NOIS-SSI database. Not classified as a registry but has some registry features. | - During the first 5 years of operation, NOIS has achieved almost complete hospital participation, a reasonable proportion of non-missing explanatory variables and 90.7% completeness of 30-day follow-up of patients  (mean time until post-discharge infection detected was 13.2 days (95% confidence interval: 12.7-13.8).  - Found 81% of infections were detected after discharge from hospital.  - Concluded active post-discharge surveillance is important and will detect many infections that would otherwise remain undetected. | - Important success factors are a mandatory system, automated  data-harvesting systems in hospitals, and active post-discharge surveillance.  - Computerised harvesting of data from existing electronic hospital systems is feasible and eases burden on staff.  - Infection control staff can generate reports and statistics from the computerised infection control module (ICM), which can be used in feedback to the surgical departments and hospital management and for quality control.  - ICMs simplify submission of data to the national level by generating data files in specific formats.  - Data quality can be improved through checks and subroutines that are programmed into ICMs. |

| **First author, location, year** | **Study Objectives, Benchmarking?** | **Type of registry** | **Healthcare findings** | **What works?** |
| --- | --- | --- | --- | --- |
| Nag  Australia  2019 | - To determine acceptability and feasibility of providing structured feedback in addition to the regular performance feedback reports to key personnel with intent to drive quality improvement.  - Registry provides feedback on key performance indicators (KPIs) through comparison to other participating Units. | The Australian and New Zealand Society of Cardiac and Thoracic Surgeons (ANZSCTS) Database | - Survey response to current feedback report: 95% agreed content is relevant; 85% thought the KPIs were useful; and 75% felt it would be beneficial to compare surgeons’ KPIs.  - Survey responses to method of feedback: 67% of control units requested structured feedback sessions one to two times annually; and 83% of intervention units requested future structured feedback.  - Limitations of feedback in driving QI was high performance of units at baseline, low surgeon participation, and scheduling challenges for structured feedback.  - Structured feedback **did not** significantly improve communication.  - Quarterly reports are provided to Heads of Units and Data Managers for dissemination to the Unit’s multi-disciplinary surgical team at the discretion of the Head of Unit. | - Structured feedback improved performance on one KPI in one outlier unit between the two sessions -held 10 months apart.  - Quarterly report distribution should be expanded to include all participating surgeons within the Unit. |
| Norman  Sweden  2020 | - To investigate the mechanisms that lead various clinicians to use quality registers for improvement. | The Swedish Heart Failure Register  (SwedeHF). | - Few new initiatives in the studied program reached the clinical context. Authors explain this through the lack of an organisational improvement logic to counter the clinician logic.  - Apparent division between the improvement leaders and the medical doctors.  - Although they had both used SwedeHF, they talked about the register and its benefits differently and used different expressions, which reinforced the gap between them.  - A strong path dependence was identified, as registers have historically been tightly linked to the medical profession’s competence.  - Medical doctors’ influence is manifested through both passive and active ways of restricting [registry] activities.  - In the registration context there is the professional division in practice which has prevented learning among professionals.  - Within the governance structure, policy officials and administrators face difficulties of implementing new ideas as the medical profession opposes these initiatives.  - The introduction of pay for performance has led to an increased focus on NQRs and also to increasing competition between registers. | - Need for professionals who can bridge these different rules, so called hybrid professionals. They are representatives of the medical profession, who relate not only to their clinician logic but also to the  logic of organisational improvement.  - Development of healthcare systems would benefit from finding ways how to identify and place hybrid  professionals in leading positions. |
